# Global tastes, local choices: Strontium isotope and concentration evidence for changing dietary input in Roman Nijmegen, the Netherlands

**DOI:** 10.1371/journal.pone.0349604

**Published:** 2026-05-26

**Authors:** Maura De Coster, Joep Hendriks, Hannah F. James, Christophe Snoeck, Gareth R. Davies, Lisette M. Kootker

**Affiliations:** 1 Vrije Universiteit Amsterdam, Faculty of Science, Department of Earth Sciences, Geology & Geochemistry cluster, Amsterdam, the Netherlands; 2 Gemeente Nijmegen, Bureau for Archaeology, Nijmegen, the Netherlands; 3 Archaeology, Environmental Changes & Geo-Chemistry, Vrije Universiteit Brussel, Brussels, Belgium; 4 Vrije Universiteit Amsterdam, Faculty of Social Sciences and Humanities, Department of Art and Culture, History, and Antiquity, Antiquity cluster, Amsterdam, the Netherlands; University of Padova: Universita degli Studi di Padova, ITALY

## Abstract

This study investigates dietary practices and local interaction in Roman and pre-Roman Nijmegen, the Netherlands, through strontium isotope ratios (⁸⁷Sr/⁸⁶Sr) and concentration measurements on cremated human remains from rural, urban, and military contexts, which primarily reflect the geological provenance of dietary strontium. Located along the Lower Rhine borderlands, Nijmegen formed a key node within the Batavian region, where local communities intersected with imperial networks. The results reveal isotopic contrasts between site types: urban and military groups tend to display higher strontium concentrations, possibly consistent with greater engagement with salt-preserved and salt-rich foods, while in the northern contexts relatively stable concentrations alongside a narrowing of isotope ratios may suggest continuity in dietary practices, potentially including earlier traditions of salt consumption. These patterns indicate that dietary change was not uniform, but shaped by the interaction between emerging imperial foodways and pre-existing local practices. Rather than reflecting simple differences in access, the isotopic evidence points to varied and context-dependent forms of engagement with Roman provisioning systems.

## 1. Introduction

Globalisation theory, though modern, has been widely applied to the Roman Empire to explain how distant regions became interconnected through trade, migration, imperialism, and the exchange of goods, ideas, and cultural practices. Globalisation has also been introduced as a good alternative to the Romanisation model, since it moves away from the top-down and centre-periphery approach by emphasizing mutual influence and local agency, and it also holds space to discuss dissonance, uneven integration and resistance besides adoption. Since the introduction of the globalisation concept into Roman archaeology, many researchers have adopted it as a framework to discuss their findings on trade, cultural practices, and mobility [1–4].

In parallel with this theoretical shift from Romanisation to globalisation, methods for studying mobility in the Roman world have also developed significantly. Whereas early archaeological approaches often focused on the movement of objects, more recent work increasingly examines the movement of people and the circulation of ideas [5–9]. This has been supported by the growing use of analytical techniques such as aDNA, proteomics, network analysis, and especially isotope analysis. Strontium isotope analysis (^87^Sr/^86^Sr) has become an important tool for identifying ‘non-local’ individuals and reconstructing patterns of mobility. Introduced in archaeology in the 1980s with Ericson’s work on “dietary input from discrete geochemical environments” [10], the method is now widely applied to investigate human and faunal movement, among other applications (e.g., [6,11–16]).

While studies on mobility and migrations over large geographical areas have proven to be immensely valuable [6,16–19], this study aligns with a growing body of research within archaeological science that advocates for smaller-scale perspectives, with greater attention to the complexities of archaeological theory, methodology, and archaeological context [5,11,20,21]. Globalisation theory, with its emphasis on connectivity, mutual influence, and uneven integration, offers a productive framework for interpreting such localised interaction. Applied on a micro-scale, it helps illuminate how everyday movements contributed to and were shaped by wider exchange networks. Case studies of local contexts have shown the potential of this approach, providing more nuanced understandings of cultural and economic exchanges during the Roman period [21–25]. Moreover, the strontium isotope system is applied almost exclusively as a proxy for human mobility. However, human dietary ^87^Sr/^86^Sr primarily reflects the provenance of dietary resources and, in some particular cases, it is also possible to gain insights into specific types of foods consumed such as salt by combining strontium isotopes and concentrations [26]. Nevertheless, diet is an aspect still rarely considered in the interpretation of human strontium isotope data, although new studies from, among others, Roman Europe underline its importance within mobility research [27,28].

Building upon the insights of globalisation theory regarding interconnectedness and exchange in the Roman Empire and of the complexity of dietary human ^87^Sr/^86^Sr, this study employs strontium isotope (n = 331) and elemental analysis (n = 221) on cremated human individuals from well documented rural, urban and military Roman and pre-Roman rural cemeteries in the Nijmegen region in the Netherlands. During the Roman period, the present city of Nijmegen lay at the heart of the Batavian region, strategically positioned along the river Waal, a branch of the Rhine. The Rhine itself marked the northern border of the Roman Empire, the limes, making Nijmegen a key hub along this border zone. To the south of the Waal, Nijmegen served as a major Roman urban centre, *Ulpia Noviomagus Batavorum*, while to the north of the river lay a series of smaller rural settlements. This paper shows how globalisation theory and strontium isotope and elemental analysis can contribute to the understanding of diet in relation to daily life and evaluates the potential of a micro-scale approach in archaeological isotope research.

## 2. Archaeological background

**Fig 1:**
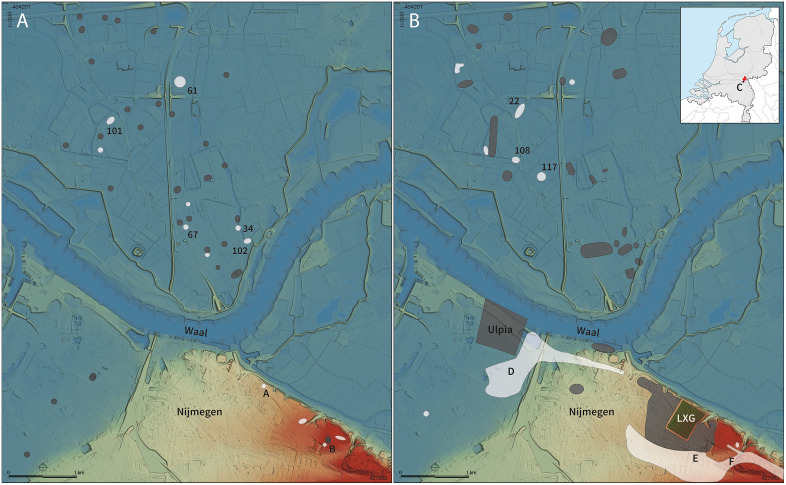
Nijmegen in the (A) Early/Middle Iron Age & (B) Middle Roman period, with a selection of the main settlements (grey), the sampled cemeteries (white) and the fortress of the Tenth Legion (dark green, LXG). The sites corresponding to the numbers and letters can be found in Table 1. The background is based on data from the Current Height Model of the Netherlands [38] (under a CC BY license). Heumen (site **C**), located circa 8 km south of Nijmegen, is indicated on the inserted map.

In late prehistory, the area around present-day Nijmegen was highly attractive for habitation. The alluvial landscape to the north of the river Waal, known as Nijmegen-North, consisted of residual channels and sandy levees and has been inhabited continuously since at least the second half of the Middle Bronze age (c. 1500–1100 BCE). Judging by the several burial sites that have been discovered in the past two decades, the number of settlements –hamlets comprising one to three farmhouses and some granaries– must have increased significantly during the subsequent Early Iron Age (c. 800–500 BCE) [29,30]. Within an area of approximately 13.5 km^2^, at least seven cemeteries including cremations and inhumations (dating back to the Early Iron age) have been identified, five of which were used for some time during the Middle Iron Age (c. 500–250 BCE) (Fig 1A).

During the 5^th^ century BCE, several richly furnished elite cremation burials appeared in the Nijmegen area south of the Waal and in the neighbouring village of Heumen. Two small cremation cemeteries were established on the high ridge of the outwash plain and moraine (Fig 1A), a burial landscape already in use for more than a millennium. Several cremation graves at the Kops Plateau contained iron arrowheads and spearheads, while a cremation grave at Traianusplein held the dismantled remains of a vehicle. In nearby Heumen, the cremation grave at Hessenbergseweg was furnished with weaponry, chariot components, and drinking equipment. Together, these burials point to the integration of the region into supra-regional elite networks at the beginning of the Middle Iron Age [31,32].

During the Late Iron age, following a demographic decline, the arrival of the Roman army in the Nijmegen region marked a new period of settlement growth. In the Early Roman period (from 19 BCE to the Batavian Revolt in 69/70 CE), several military complexes and a proto-urban centre were constructed south of the Waal (Fig 1), including the three successive fortifications on the Kops Plateau (12 BCE–69/70 CE). After the revolt, the topography of Roman Nijmegen underwent significant changes, with the construction of the *Legio X Gemina* fortress (72–103/104 CE) and its *canabae legionis* on the elevated outwash plain, as well as the Roman town and Batavian capital *Ulpia Noviomagus* (c. 70/100–280 CE) in the low-lying river plain to the west [33,34]. To the east and south of the legionary fortress, a (semi-)military cemetery emerged: here, the Kruisweg site had already been in use since 12 BCE, but other parts, such as the Hengstdalseweg site, were only used after the arrival of the Tenth Legion, shortly after 69/70 CE (Fig 1B). The installation of the new Roman town on the south bank of the river Waal resulted in the development of a similarly sized cemetery (Onder Hees) for the urban inhabitants in Nijmegen-West. Notably, the first generation of cremation burials at the Krayenhofflaan comprises several richly furnished elite graves [35]. From the late 2^nd^ century, the inhumation rite gradually became more common into urban Roman Nijmegen society.

North of the river Waal, the local population under Roman rule gradually grew from a few hamlets in the early 1^st^ century CE to at least ten settlements by the mid-2^nd^ century CE (Fig 1B). During this period, the local rural communities became integrated into provincial-Roman society as a result of their service in the Roman army and their obvious contact with the nearby Roman town and fortress [36]. Numerous cemeteries must have existed in the Nijmegen-North area, but only five have been extensively investigated. While some inhumations are present at the Van Boetzelaerstraat and Rust Wat sites, the cemeteries are dominated by cremations, which is in line with regional burial customs. Only a few cremation graves stand out, with more elaborate grave constructions and grave gifts, demonstrating a clear understanding of the Roman burial practices which were particularly common among urban and villa elites [37].

When interpreting the data from these sites, the heterogeneous chronology of the assemblage must be taken into account. The Late Iron Age is entirely absent from the dataset, as no settlement or burials of this period had been identified at the time of this study. The Iron Age graves that were sampled span multiple centuries, covering a broad temporal range. In addition to this chronological breadth, the funerary record reflects considerable variation in population composition: the Iron Age graves south of the river represent elite cremation burials, whereas those north of the river are associated with to small hamlets. For the Roman Period similar observations can be made. For the purposes of this study, the Roman period populations are divided into three categories based on their archaeological context: urban, rural and military. The urban groups represent an elite stratum of Roman urban society and, like their Iron Age counterparts, were interred south of the river. The rural cemeteries are situated north of the river, while the military cemeteries, the earliest among the Roman groups, were located south of the river. The chronological range and contextual diversity of these assemblages must therefore be kept in mind when interpreting the data. A timeline indicating the dates of use for each cemetery is provided in Table 1.

**Table 1:** Overview of the archaeological sites from Nijmegen and Heumen and the number of samples included in this study.

							N	
Period	Location	Site	Toponym	Code	Type of site	Occupation phase	Individuals	Sampled individuals
Iron Age	North of Waal	101	Eeuwige Lente	Bo5	Rural	BCE 775-550	32	6
		34	Laauwikstraat	La2		BCE 550-400	9	3
		67	Lentseveld	Lv9		BCE 650-500	12	7
		102	Stelsestraat	Sl2		BCE 650-500	32	8
		67	Zuiderveld-noordoost	Zv10/Zn3		BCE 650-250	35	9
	South of Waal	A	Traianusplein	ROB 1974	Elite	BCE 500-400	5	3
		B	Kops Plateau	ROB 1986–1995			10	5
		–	Hessenbergseweg	RCE 2018			1	1
Roman	North of Waal	22	Griftdijk/‘t Klumke	Ngk2–9	Rural	CE 50-225	26	26
		128	Van Boetzelaerstraat	Bo6/8		CE 50-225	100	28
		117	Broodkorf	Nlz9/13_Brk1		CE 50-110	10	5
	South of Waal	D	Voorstadlaan/Krayenhofflaan	Vr8/KUN	Urban	CE 80-270	102	62
		E	Hengstdalseweg	Hd1–2	Military	CE 70-120	58	16
		F	Kruisweg	Kw1		BCE 12-CE 120	177	12
**TOTAL**							**609**	**191**

## 3. Material

### Site selection

#### Early and Middle Iron Age (BCE 800–250).

Early (BCE 800–500) to Middle (BCE 500–250) Iron Age cremation graves from Nijmegen Eeuwige Lente [39], Laauwikstraat-Zuid [unpublished], Lentseveld [40], Steltsestraat [41], and Zuiderveld [42,43] were selected and sampled as part of the ‘Travelling Bands’ project which aimed to develop and validate a novel research methodology for investigating the connection between provenance and grave use [44]. These sites are all located north of the river Waal, i.e., in Nijmegen North. In addition, published data from Middle Iron Age Nijmegen south of the river Waal (Traianusplein and Kops Plateau), and the village of Heumen, south of Nijmegen, that were generated within the framework of the interdisciplinary ‘Chariots of Fire’ project on early La Tène elite burials from the Lower Rhine-Meuse region and their Northwest European context are included in this study [31] (Fig 1). As both projects focused on the presence of high status or elite burials, the Iron Age sites included in this study were biased towards the presence of one or more graves equipped with grave goods. A complete overview of the sites, number of individuals and samples, and associated (radiocarbon) dates is provided in Table 1.

#### Early and Middle Roman period (BCE 19–250 CE).

Given the extensive number of Roman cremation graves in Nijmegen, any selection only represents a very small fraction of the city’s Roman population. Despite efforts to achieve a representative sample of sites, the selection is inevitably influenced by factors such as cemetery type, the extent of archaeological investigation and documentation, and the preservation quality of the cremated remains. First, based on the (published) archaeological interpretation of the sites and their associated finds, the cemeteries were roughly divided into three categories that are thought to accurately represent the diverse population of *Noviomagus*: rural, urban, and military. All sites assigned to the first category are in Nijmegen North, while the latter two categories represent sites situated in the sandier regions south of the river (i.e., Nijmegen South). Next, within these categories, cemetery sites that were well-documented and researched were prioritised over those with incomplete documentation. Finally, the overall preservation of the human remains was considered, particularly focusing on the weight of the cremated remains and the degree of burning, i.e., the presence of fully calcined bones to ensure the presence of biogenic ^87^Sr/^86^Sr [45]. This approach resulted in the selection of five rural (‘t Klumke [46], Rietgraaf [47], Van Boetzelaerstraat [unpublished], Rust Wat [48], and Broodkorf [49]), one urban (Onder Hees [35]), and two military sites (Kruisweg and Hengstdalseweg [50]) (Table 1).

### Sample selection

#### Human samples.

The various sampling campaigns aimed to select three different skeletal elements per cremation: a long bone diaphysis (n = 165), a rib fragment (n = 135) and a tooth root (i.e., dentine, n = 31). Petrous elements were present in the assemblage but were excluded from the analysis. In all cases, preservation was insufficient, either due to incomplete cremation or fragmentation, to yield viable samples. This sampling strategy leverages the differing bone turnover rates of each skeletal element, offering a unique opportunity to reconstruct individual life histories, as each element reflects distinct periods of life (see De Coster et al. [51] for a detailed discussion). For the main statistical analyses, only diaphysis samples were included to ensure consistency across individuals and to avoid counting the same individual multiple times. Rib and dentine data are provided in the Supplementary Material and discussed separately in relation to the diaphysis results. In short and depending on the estimated age-at-death, the selected skeletal element, sample location within that element, and general health, among other factors, the ^87^Sr/^86^Sr of the cortical bone of the long bones is thought to reflect the dietary Sr intake during the last years to decades of life. In contrast, due to its faster bone turnover rate, cortical, but specifically trabecular rib bone ^87^Sr/^86^Sr should be indicative of that of the dietary intake during most recent years prior to death [52]. Consequently, a change in geolocation or in (the origin of the) diet during life may be reflected in isotopic differences between the different samples [53]. Although in theory individual life histories can be reconstructed in this way, one must account for a known practice of the past, of depositing cremated remains from multiple individuals within a single grave. Such mixing can create the illusion of intra-individual mobility, when in reality the observed variation reflects isotopic signatures from different individuals [53–56]. To minimise the risk, only graves with a minimum number of individuals (MNI) of one were included. Graves in which the possible presence of more than one individual was indicated (e.g., on the basis of duplicate skeletal elements, anomalously high bone weight, or marked variation in cortical robustness) were excluded from sampling. Despite these precautions, the possibility of inadvertent commingling cannot be entirely ruled out.

In practice, for most individuals a diaphysis and a rib were the only skeletal elements that were calcined (minimum burn degree IV, conform Lemmers [57]) and therefore eligible for Sr isotope analysis [38]. This process resulted in the selection of 194 individuals (51 Iron Age, 143 Roman period) excavated within the Nijmegen region (Table 1). It must be noted that even though specific care was taken to sample the Roman rural, urban, and military sites proportionally to the approximate number of graves each category entailed, a smaller number of samples was selected for the military category due to their generally less extensive documentation compared to the other categories. All of the samples were collected from the archaeological depot of Nijmegen, province of Gelderland. No permits were required for the described study, which complied with all relevant regulations.

#### Vegetation samples.

To enhance the existing reference dataset and enable more accurate interpretation of human ^87^Sr/^86^Sr, 33 modern vegetation were sampled and incorporated into the baseline alongside previously published data [56,58–60]. Where possible, vegetation with varying root depths, i.e., trees, shrubs, and crops, was sampled at each location. The sampling sites were selected to represent the full range of geological and/or palaeogeographical subsurfaces in the region, ensuring that even with a relatively low sampling density, the dataset provides a reliable approximation of the bioavailable ^87^Sr/^86^Sr signatures in the Nijmegen area.

## 4. Methods

### Sample preparation

#### Human cremated remains.

A detailed overview of the applied sample preparation and leaching protocol is provided in De Coster & Kootker [51] and summarized here. If possible, subsamples of the selected bone fragments were taken and mechanically cleaned by removing the outer layer and any remaining sediments with a Proxxon diamond coated ball burr. Next, the cleaned bone fragments were transferred to glass vials and leached with circa 1.0 mL (10:1 ratio) 1M acetic acid (CH_3_COOH) ultrasonically for 3–10 minutes, followed by two Milli-Q™ rinses, and a 10-minute Milli-Q™ ultrasonic wash [45,61]. These steps were repeated until the Milli-Q™ in the glass vials was clear or showed a white cloudy colour (see Fig 2 in [51]). All samples were dried on a hotplate at 50 °C overnight, powdered, and transferred into 1.5 mL capped glass vials for further analysis.

**Fig 2:**
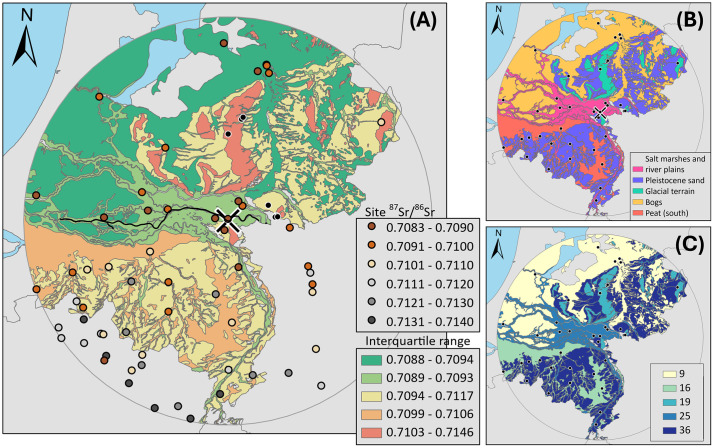
Bioavailable Strontium Baseline around Nijmegen. **(A)** Multi-proxy bioavailable strontium baseline for the modern-day Dutch territory within 100 km of Nijmegen over the 100 CE palaeogeography map [65] (under a CC BY license, from Rijksdienst voor het Cultureel Erfgoed). Map colours defined by the interquartile range (Q1 to Q3) of all samples within each surface geological description category for the Netherlands. Location of the Waal River indicated by black line **(B)** Surface geological categories from the 100 CE palaeogeography map used as a basis of the A map. **(C)** Number of samples within each surface geological description category. Country boundaries are sourced from [69] under a CC BY license, from William & Mary geoLab.

#### Vegetation.

Approximately 300–400 mg of each sample was weighed into acid-cleaned PTFE vessels (MARS 6 microwave system, CEM Corporation, North Carolina, USA) and digested in a solution of 8 mL of 14M HNO_3_ and 3 mL of ultrapure H_2_O_2_. Once fully dissolved and transparent, the solutions were transferred to pre-cleaned 15 mL Savillex® beakers and evaporated to dryness. To ensure complete digestion, 14M HNO_3_ and ultrapure H_2_O_2_ were added and the samples were re-evaporated until no solid residue remained.

### Analytical details

#### Sr isotope composition.

The strontium purification process was carried out in the same clean laboratory facility at the Vrije Universiteit Amsterdam. The fully digested vegetation samples were dissolved in 1.0 mL 3M HNO_3_, transferred to acid-cleaned Eppendorf® tubes and centrifuged for 3 minutes at 12000 RPM. The dried bone powder was weighted into acid-cleaned Eppendorf® tubes and dissolved in 0.6 mL 3M HNO_3_, and centrifuged. Next, 0.5 mL was put over in-house made 2 mL pipette-tips columns filled with 80 μL Eichrom Sr resin (100–150 μm mesh) to extract the strontium [51].The purified strontium samples were collected in HCl-HNO_3_ cleaned 7 mL Savillex® Teflon vials with conical interior and nitrated with 14M HNO_3_. The blanks were spiked with an ^84^Sr enriched in-house made solution.

The dried purified Sr samples were redissolved in 2 μL 10% HNO_3_ and 1 μL was loaded onto single annealed rhenium filaments with 2 μL TaCl_5_. A Thermo Scientific^TM^ Triton Plus^TM^ thermal ionisation mass spectrometer (TIMS) at the Vrije Universiteit Amsterdam was used to determine the isotope compositions using a static routine. A total of 15 vegetation samples from the Netherlands (see supplementary data) were microwaved and measured at the Vrije Universiteit Brussel using a Nu Instruments Plasma III Multi-Collector Inductively-Coupled-Plasma Mass-Spectrometer (MC-ICP-MS; PD017), following the procedure in Gerritzen et al. [62]. All Sr isotope ratios were corrected for mass fractionation to ^86^Sr/^88^Sr = 0.1194. During this study (2022–2024), the reproducibility of the TIMS was 0.710259 ± 0.000017 for repeated analysis of NIST® SRM® 987 (n = 267, 2s, loading size 200 ng). This is comparable to the long-term reproducibility (2017–2024): 0.710256 ± 0.000018 (n = 696, 2s). NIST® SRM®1400 and 1515 were also continually analysed during this study and resulted in a reproducibility of respectively 0.713108 ± 0.000028 (n = 20, 2s) and 0.713960 ± 0.000020 (n = 15, 2s). Details on the reproducibility of the MC-ICP-MS are published in Sengeløv et al. [60]. The ^87^Sr/^86^Sr are presented with a margin of error of two standard errors (2SE).

The column blanks (n = 17) contained an average of 28 pg Sr. This amount is considered negligible when compared to the average amount of strontium present in cremated bone, faunal enamel, and vegetation samples [45,61,63,64].

#### Concentration measurements.

Aliquots were taken from the remaining cremation samples in the 0.1 mL 3M HNO_3_ solution and diluted to a total dilution factor of 10,000 in acid-cleaned ICP vials. The strontium ([Sr]) and calcium ([Ca]) concentrations were measured on a ThermoFisher iCAP TQ ICP-MS. Sr (m/z = 88) was measured no gas in the collision-reaction cell, while kinetic-energy-discrimination (KED) using helium (He) was applied for Ca (m/z = 44). Indium (In) was used as an internal standard to correct for sensitivity drift, and quantification was achieved via external calibration against calibrants gravimetrically prepared from certified single-element standards. The NIST® 1400 bone-ash SRM was used for quality control. Repeated digestions and measurements of the NIST® SRM® 1400 (n = 13) yielded recoveries of 89.6–105.9% for Ca and 88.9–101.3% for Sr, with intermediate precision (i.e., repeatability of individual analyses) better than 5% relative standard deviation (1s). All [Sr] were normalised to 40% Ca to account for organic loss using the following equation:


$$\begin{array}{c} {\left[Sr\right]}_{normalised}=\frac{{\left[Sr\right]}_{measured}}{{\left[Ca\right]}_{measured}}\;\times \;4\text{0}\% \end{array}$$

### Mapping

The newly generated modern vegetation data were combined with both published and previously unpublished ^87^Sr/^86^Sr from archaeological rodents [59], alongside previously published plant data [56,58,60] to characterise the regional bioavailable strontium signature. In total, 167 modern vegetation and archaeological rodent samples from 62 sampling sites within a 100 km radius of Nijmegen were used to establish the baseline. For the Netherlands, 105 samples are used to the create the baseline, with 23 samples from Germany and 39 samples from Belgium used to establish the wider regional ^87^Sr/^86^Sr signature (Fig 2). All data used to construct the bioavailable strontium baseline are publicly available on the IsoArcH database (DOI 10.48530/isoarch.2025.013) and can be found in the supplementary data (S1 File). As the Dutch landscape has undergone significant change over the last two thousand years, a palaeogeographic map depicting the landscape in 100 CE, which also closely resembles that of the Iron Age in the Nijmegen region, was used as a base map [65,66]. In addition, the Dutch soil map [67] was used to distinguish between raised bog and fens (referred to as ‘peat north’ and ‘peat south’, respectively), since their differing modes of formation lead to significant differences in the ^87^Sr/^86^Sr of modern vegetation. As such, it provides a suitable framework for visualising and interpreting bioavailable strontium distributions relevant to both periods. To capture regional differences in peat within the Netherlands, this category was split into north and south regions along the Amer River.

**Fig 3:**
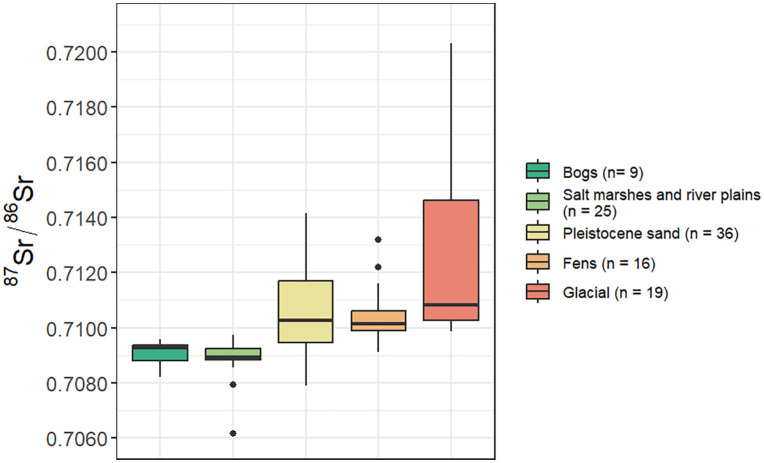
Boxplots of the distribution of ^87^Sr/^86^Sr for each description category from within the Netherlands. Data from sites located within Belgium and Germany not included.

Spatial summary statistics and baseline creation were undertaken in ArcGIS Pro 2.9.2 using the Spatial and Geostatistical Analyst extensions (ESRI), and statistical analysis and graph creation undertaken in R Studio using ggplot2 package. For the Dutch baseline, the 100 CE paleogeographic map was spatially joined with the ^87^Sr/^86^Sr sampling site data, providing a description of the underlying geological surface for each site. To create a prediction for ^87^Sr/^86^Sr away from sampling sites in the Netherlands a domain mapping approach was employed [68]. Predictions are based on shared geological characteristics, here the surface geological categories from the 100 CE paleogeographic map. All ^87^Sr/^86^Sr measurements within each surface geological category were then used to determine the category’s interquartile range (Q1 to Q3) (Fig 3A and 3B). To allow for the assessment of the robustness of this prediction the number of ^87^Sr/^86^Sr samples within each category is also presented (Fig 2A). Including the number of samples within each geological surface category provides an indication of how well the prediction is supported by data. Categories with higher sampling density are more statistically robust, while categories with fewer samples have greater uncertainty and more susceptible to outliers and sampling bias.

### Statistics

Statistical analyses were conducted in IBM SPSS Statistics (v.30). Non-parametric Mann–Whitney U tests were used to assess differences between groups, with effect sizes (*r*) reported. A one-way ANOVA on normalized [Sr] values with planned contrasts was applied for multi-group comparisons.

## 5. Results

### Sr isotope and [Sr] data from cremated human remains in Nijmegen

**Fig 4:**
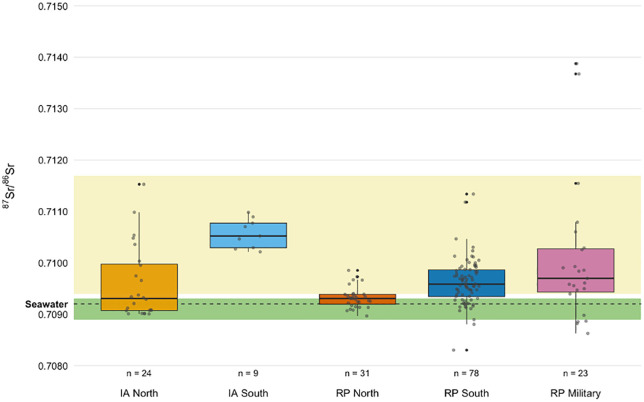
Boxplots of ^87^Sr/^86^Sr measured in long bone diaphyses grouped by region and period: Iron Age (IA) North, IA South, Roman Period (RP) North, RP South, and RP Military. Shaded bands indicate the estimated local bioavailable ^87^Sr/^86^Sr ranges for the northern (green) and southern (yellow) regions. The dashed line denotes the seawater ^87^Sr/^86^Sr of 0.7092 ([57]), included for reference; this ratio falls within the estimated local bioavailable ⁸⁷Sr/⁸⁶Sr range of the northern region.

When the Iron Age dataset is divided into two groups, rural individuals living north of the Waal (n = 24, ranging from 0.7090 to 0.7120; IQR 0.7090–0.7100, 0.0009) and elite graves from the population residing south of the Waal (n = 9, ranging from 0.7102 to 0.7110; IQR 0.7104–0.7108, 0.0005), the southern group plots within their bioavailable baseline, whereas the northern group overlaps with both the northern and southern baseline (0.7089–0.7093 and 0.7094–0.7117 respectively). The median of the northern groups coincides with the upper limit of the northern bioavailable baseline (Fig 2). The northern group plots toward the higher end of its baseline range, whereas the southern group plots near the middle of the yellow band in Fig 4. The difference in population size between both Iron Age groups is uneven due to sample availability.

The Roman rural dataset from north of the Waal (n = 31) displays a narrower distribution of ⁸⁷Sr/⁸⁶Sr than the Iron Age north population, despite a comparable median, with ratios varying from 0.7090 to 0.7099 (IQR 0.7092–0.7094, 0.0003), situated between the two baselines. A Mann-Whitney U test also showed no statistically significant difference (U = 389, p = .934). In contrast, the Roman urban population from the south (n = 78) shows similar variability, yet lower ^87^Sr/^86^Sr (0.7090–0.7113, IQR 0.7094–0.7099, 0.0005) than the southern Iron Age population, despite residing in the same geological region. This was confirmed to be a statistically significant difference between the ^87^Sr/^86^Sr from IA South and RP South by a Mann–Whitney U test (U = 22, p < .001). Additional Mann–Whitney U tests were conducted to assess differences in ^87^Sr/^86^Sr between the IA and the RP rural (north) and urban (south) groups. Significant differences were found between the northern and southern groups in both the Iron Age (U = 167, Z = 3.09, p < .001, r = .55) and the Roman period (U = 1813, Z = 3.72, p < .001, r = .36). The r-values, which represent the practical significance of the differences, indicate a large effect in the Iron Age that decreases to a medium effect in the Roman period.

The dataset derived from the military population (n = 23), whose cemeteries were also located south of the river Waal, exhibited the greatest variability within the Roman population, with ^87^Sr/^86^Sr ranging from 0.7086 to 0.7139 (IQR 0.7095–0.7106, 0.0011).

**Fig 5:**
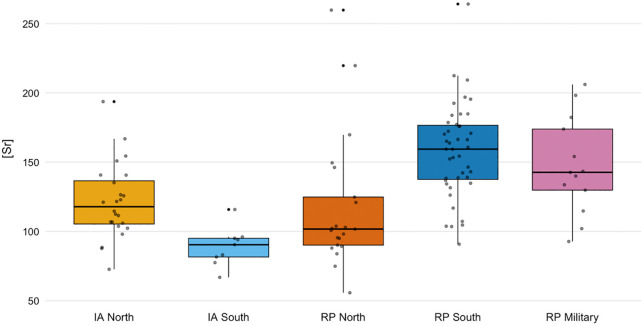
Boxplots of strontium concentrations ([Sr] in ppm, normalised to 40% Ca) in human long bone diaphyses grouped by region and period: Iron Age (IA) North, IA South, Roman Period (RP) North, RP South, and RP Military. Several individuals were identified as outliers in both the ⁸⁷Sr/⁸⁶Sr and normalized [Sr] datasets. Within the Roman-period military group, two individuals display markedly elevated ⁸⁷Sr/⁸⁶Sr relative to the rest of the dataset: G29 P5 (0.7139) and G26 P11 (0.7139). The Roman period southern urban group contains three outliers: two with elevated ratios (0.711180 and 0.711338) and one with a low ratio (0.7083). In the Roman period northern rural group, two individuals fall outside the main isotopic cluster (0.7099 and 0.7097). One individual from the Iron Age northern group (Graf 4) is an outlier in both datasets, exhibiting an elevated ⁸⁷Sr/⁸⁶Sr (0.7115) and the highest normalized [Sr] value recorded in the assemblage (193.7 ppm). Elevated concentration outliers are alse present in the Roman period northern rural group (259.7 and 219.6 ppm) and in the Roman period southern urban group (264.1 ppm).

The [Sr] from the Iron Age population ranges between 73–194 ppm, with the northern (IQR 104–138, 35 ppm) Iron Age populations exhibiting a higher [Sr] range than the southern (IQR 82–96, 14 ppm) population (Fig 5). A Mann-Whitney U test (U = 27, Z = −3.003, p = .003, r = .53) showed a statistically significant difference between the Iron Age groups. In the Roman period, the populations living on the different banks of the river also exhibit different [Sr]. The southern urban population has a slightly more variable and higher [Sr] (range 90–264 ppm, IQR 138–177, 40 ppm) than the rural population living North of the Waal (range 56–260 ppm, IQR 90–125, 46 ppm). This was shown to be a statistically significant difference by a Mann-Whitney U test (U = 780, Z = 4.26, p < .001, r = .53). The [Sr] from the early Roman period military population varied between 93–206 ppm (IQR 130–174, 56 ppm). Further Mann-Whitney U tests showed that there is no statistical difference between the Iron Age and Roman Period groups north of the Waal (U = 181, p = .068), whereas the [Sr] from the southern groups exhibited a statistically significant difference (U = 336, p < .001) between the Iron Age and the Roman Period. A one-way ANOVA confirmed a significant group effect on normalized [Sr] (F(2,74) = 7.66, p < .001). Planned contrasts with the military group as reference indicated that the military group differed significantly from the northern group (p = .033) but is statistically indistinguishable from the southern group (p = .388). Thus, the military group more closely resembles the southern population than the northern one. All datapoints included in this study are reported in more detail in the Supplementary Data (S2 File) and can be retrieved from the IsoArch repository DOI 10.48530/isoarch.2025.013.

**Fig 6:**
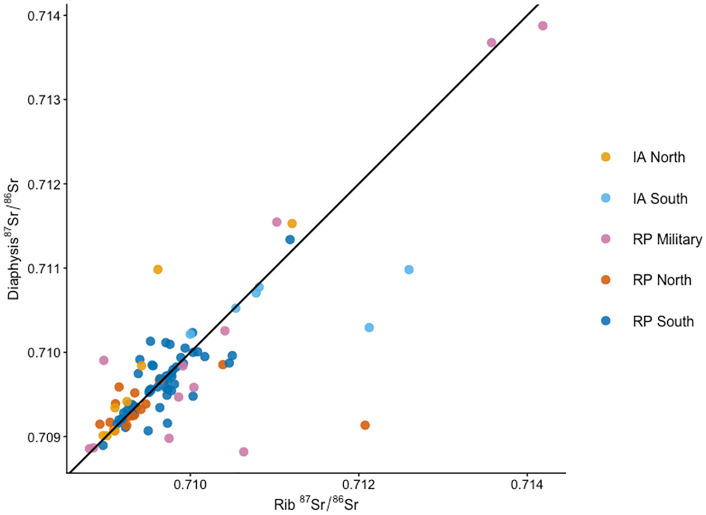
Scatterplot of diaphysial versus rib ⁸⁷Sr/⁸⁶Sr for individuals with paired samples, grouped by region and period: Iron Age (IA) North, IA South, Roman Period (RP) North, RP South, and RP Military. The diagonal line represents perfect agreement between the two skeletal elements. Points plotting close to this line indicate no isotopic difference between the diaphysial and rib samples, whereas deviations may reflect individual mobility or biological differences in strontium uptake between tissues formed at different life stages.

Figure 6 compares ⁸⁷Sr/⁸⁶Sr measured in rib and long bone diaphyseal samples from the same individuals. Most paired measurements plot close to the 1:1 line, indicating a strong correspondence between isotopic ratios obtained from the two skeletal elements. The majority of individuals cluster within a narrow isotopic range between approximately 0.7090 and 0.7105 in both tissues, consistent with the dominant range observed across the wider dataset. Several individuals deviate from the 1:1 line (G126, G24 P13, Graf 7, G79, Graf 60/9, G26), displaying higher or lower strontium isotope ratios in one skeletal element relative to the other. These deviations occur across multiple groups but are most pronounced in a small number of individuals within the Roman period military and southern urban groups. Individuals from the Iron Age northern and Roman rural populations generally show closer agreement between the two skeletal elements. Overall, both skeletal elements yield broadly comparable ⁸⁷Sr/⁸⁶Sr for the majority of individuals; however, the deviations observed in a subset of the assemblage indicate that differences between skeletal elements do occur.

## 6. Discussion

By comparing the two distinct geological backgrounds (i.e., north and south of the river Waal: Holocene and Pleistocene sediments respectively) across the Iron Age and the Roman period, a shift in ^87^Sr/^86^Sr and strontium concentrations becomes evident. While the northern (rural) Iron age and Roman populations exhibit very similar ^87^Sr/^86^Sr, the southern populations show substantial change. In the Iron Age, the ^87^Sr/^86^Sr fall on the higher end of the for this study newly curated local bioavailable Sr baseline (Fig 3), whereas the Roman population displays significantly lower ^87^Sr/^86^Sr, shifting towards the lower end of the local baseline for Nijmegen-south. [Sr] also changes between the Iron Age and Roman Period, specifically in the southern populations, that show a significant increase. The military populations exhibits similar [Sr] to the urban southern population, whereas the [Sr] in northern populations do not show statistically significant change between periods. The isotopic shift in Sr between the Iron Age and Roman populations in Nijmegen highlights that factors beyond residential mobility must be considered. While the southern Roman group exhibits lower ⁸⁷Sr/⁸⁶Sr than their Iron Age predecessors, variability is not greater in the Roman period, as might be expected with increased mobility involving geologically diverse source areas. However, this pattern does not necessarily exclude mobility, as individuals may have originated from isotopically similar regions or within a broad local baseline. It does, nevertheless, suggest that factors such as shifts in diet and provisioning systems likely contributed to shaping the isotopic signatures.

### Salt and isotopic change

Salt and marine foods provide a crucial entry point to understanding the isotopic differences between Iron Age and Roman populations in Nijmegen. Salt was a well-established method to preserve food and has proven to play a significant role in interpreting Sr isotope ratios in the Roman Period. Bioarchaeological research in Belgium have linked elevated [Sr], in combination with a narrower range in ^87^Sr/^86^Sr approximating the value of the major source of salt, i.e., seawater (± 0.7092: [70]), to an increase in salt consumption (as salt, salted preserved foods and/or *garum* compared to the Metal Ages [27,28]. In this context, products such as *garum*, the widely used Roman fish sauce made by fermenting fish with large amounts of salt, are particularly relevant. As already noted in Dalle’s et al. & James’ et al. [27,28] research, *garum* would have contributed both salt and marine-derived strontium to the diet, further reinforcing the isotopic signal of elevated [Sr]. Despite the local Sr signature of the *civitas Batavorum,* north of the river Waal, mimicking that of seawater (0.7089–0.7093), the combination of ^87^Sr/^86^Sr with the [Sr] still makes a convincing case for a large influence of salt on the Roman Period Nijmegen diet.

**Fig 7:**
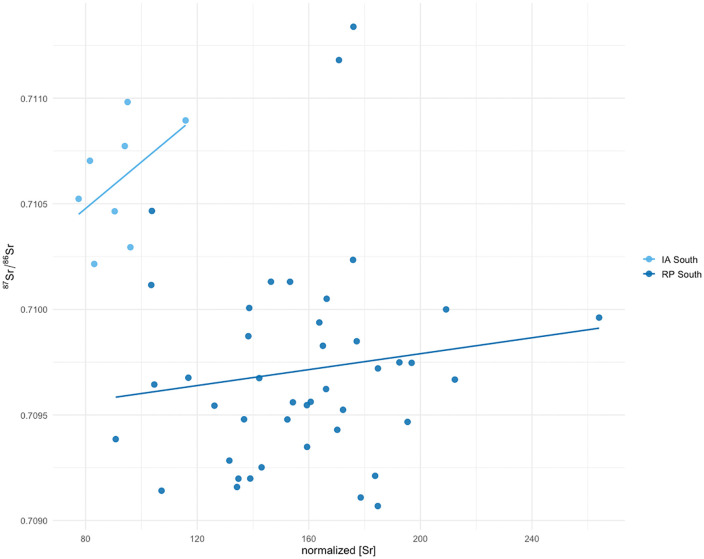
Scatterplot showing the shift of high ^87^Sr/^86^Sr and low [Sr] to lower isotopic ratios and higher concentrations, from IA to RP in Nijmegen-South.

The southern urban Roman population shows increased [Sr], and despite an increased range in ⁸⁷Sr/⁸⁶Sr a significant lower median ⁸⁷Sr/⁸⁶Sr compared to the southern Iron Age population (Fig 7). This can be explained by a greater need for food preservation, and thus salt, due to population density and Nijmegen’s role as a trade hub [71]. Archaeological evidence of salt and marine influences was found in Nijmegen as well, e.g., fish bones associated with salted fish products, fish sauce vessels and briquetage [72,73]. Such reliance on salt was not merely practical, but also reflected a broader Roman culinary tradition in which salted and fermented products, such as *garum*, played a central role [74]. The isotopic pattern in Nijmegen can therefore be understood both as a response to logistical demands and as part of the wider adoption of Roman foodways. The military population shows a different pattern: their high variability in ^87^Sr/^86^Sr, combined with [Sr] values similar to the urban group, reflects the fact that individuals that joined the army consumed food from very different geological regions and therefore likely moved between different regions. While the mobile nature of the Roman army probably did increase the need for preserved food, the precise influence of salt in their diet remains difficult to quantify.

While the range in strontium isotope ratios becomes narrower in the northern Roman period groups and shifts towards the ^87^Sr/^86^Sr of seawater compared to the northern Iron Age group, the [Sr] does not show the same clear increase as is the case in the southern populations. This difference does not necessarily reflect unequal access alone, but may reflect an earlier tradition of salt consumption. The largest collection of Iron Age briquetage pottery was excavated in Nijmegen-North and provides evidence for the presence of salt [75]. Rural households in the Iron Age may have maintained dietary traditions that relied more on salted products to prolong their food stock, whereas the Iron Age elite (south) might have been able to consume more fresh food and thus do not have elevated [Sr]. Their [Sr] are more similar to the concentration values found in other Metal Age populations in the Low Countries [27,58]. Because the northern Iron Age group may already have consumed more salt prior to the Roman period, the effect of any increase in salt consumption may be expressed primarily in the narrowing of isotopic ratios rather than in changes in [Sr]. However, this interpretation should be considered alongside alternative and potentially interacting mechanisms.

### Alternative and interacting mechanisms

Selective land use and agricultural organisation may also have contributed to the observed isotopic patterns. As suggested by De Kleijn et al., land use in the Roman-period Lower Rhine delta was structured by environmental suitability and increasing demand, with arable production concentrated on levees and pasture and meadow potentially extending into less optimal zones under pressure [76]. In this context, changes in the relative contribution of foods derived from different landscape units may have influenced dietary strontium signatures. Such processes could help explain the reduced variability and narrowing of ⁸⁷Sr/⁸⁶Sr values, particularly in the northern groups, where this pattern occurs without a corresponding increase in [Sr], [77] and may reflect more standardised or spatially constrained provisioning.

At the same time, this mechanism alone is less able to account for the patterns observed in the southern populations, which display both a directional shift toward lower ⁸⁷Sr/⁸⁶Sr values and a marked increase in [Sr]. While selective land use may influence the geological signature of dietary inputs, it does not necessarily result in increased strontium concentrations, which are shaped by multiple dietary and physiological factors. The combined nature of this shift therefore suggests that additional processes beyond land use alone may have contributed to the observed patterns. It should also be noted that [Sr] concentrations are influenced by a range of factors, including overall diet composition, the relative contribution of plant and animal products, environmental availability, and physiological processes, and therefore cannot be attributed to a single dietary component [26,78]. In addition, the use of long bone diaphysis, which reflects an averaged signal of dietary intake over the last years to decades of life, likely integrates multiple food sources and mobility histories, further complicating direct attribution to specific practices [52].

While local production likely remained important [79,80], modelling suggests that by the later Roman period the available land may have been insufficient to meet demand in all scenarios [76], potentially increasing reliance on redistribution, intensified production, or preserved foods. As such, the observed isotopic differences may reflect the interaction of multiple processes, including selective land use, changes in provisioning systems, and possibly the increased incorporation of salt-rich or marine-derived products. However, given the available data, the relative contribution of all of these factors remains difficult to disentangle. Seen from a broader perspective, these interacting processes can be situated within wider discussions of Roman-period connectivity and exchange.

### Globalising the Roman diet

Archaeologists have widely applied globalisation theory to explore social and economic structures within the Roman Empire [1–3,81]. Although globalisation is often associated with modern societies, it is equally relevant for interpreting Roman food economies and their integration of local communities.

Human diets in the Iron Age were largely subsistence-based, centred on locally cultivated grains and small-scale animal husbandry [82,83]. By contrast, the Roman period introduced new crops, diversified agricultural practices, and large-scale trade networks [79,84,85]. Their diet became more varied, supported by urban demand, military logistics, and improved storage and preservation techniques [86–89]. In this sense, salt cannot be seen as the sole driver of globalisation, but as an enabler: growing demand for Roman foodways and long-distance provisioning stimulated the expansion of salt production, which in turn reinforced the integration of Nijmegen into wider imperial markets and marked participation in Roman culinary traditions.

The isotopic evidence from Nijmegen may reflect variation in the ways broader imperial provisioning systems were manifested at the local level. Urban and military populations may have relied to a greater extent on foods brought in from elsewhere, and salt likely played a role in facilitating the preservation and redistribution of such products [74,90]. At the same time, these patterns may reflect not only economic processes, but also broader shifts in foodways: with the use of salted and fermented products forming part of wider Roman culinary traditions [86,88,89].

## 7. Conclusion

This study highlights how a micro-regional perspective can nuance broader interpretations of connectivity within the Roman Empire. In Nijmegen, differences in [Sr] and ⁸⁷Sr/⁸⁶Sr between groups may reflect variation in dietary inputs, alongside the possible persistence of pre-existing dietary practices. These contrasts, suggest that patterns associated with incorporation into wider imperial systems were not uniform, but varied according to community and context. By demonstrating how isotopic evidence can capture such variability, this study underscores the potential of bioarchaeological approaches to contribute to discussions of connectivity and food provisioning along the Roman border.

## Supporting information

S1 File BASr data for isoscape. (XLSX)

S2 File Human Data. Sr isotope and concentration human data per site.
(DOCX)
