# Supplementary material for: Global tastes, local choices: Strontium isotope and concentration evidence for changing dietary input in Roman Nijmegen, the Netherlands
Source: PLoS One. 2026 May 26;21(5):e0349604. doi: 10.1371/journal.pone.0349604 (PMC13210236; doi:10.1371/journal.pone.0349604)
Supplement: S2 File — Sr isotope and concentration human data per site. (DOCX) [file pone.0349604.s002.docx]

# Supplementary data - Sr isotope and concentration human data per site

De Coster MR, Hendriks J, James HF, Snoeck C, Davies GR, Kootker LM. ***Global Tastes, Local Choices: Strontium Isotope and Concentration Evidence for Changing Dietary Input in Roman Nijmegen, the Netherlands***

All data can also be retrieved from IsoArch: https://doi.org/10.48530/isoarch.2025.013

| **Legend** | |
| --- | --- |
| %RSD | % Relative Standard Deviation |
| normalized [Sr] | ${[Sr]}_{normalised}=\frac{{[Sr]}_{measured}}{{[Ca]}_{measured}} \times40\%$ |
| SE | Standard Error |
| Intra-ind. diff. | Intra-individual difference in ^87^Sr/^86^Sr |

All samples were obtained through the archaeological depot of Muncipality Nijmegen, province of Gelderland. The codes in the column “Individual”, refer back to the codes used by the depot.

1. **Nijmegen - Eeuwige Lente (Bo5**) [1]

| **Individual** | **Skeletal element** | **[Sr] in ppm** | **%RSD** | **[Ca] in %** | **%RSD** | **normalized [Sr]** | **^87^Sr/^86^Sr** | **2SE** | **Intra-ind. diff.** |
| --- | --- | --- | --- | --- | --- | --- | --- | --- | --- |
| Graf 6 | Longbone | 109.6 | 2.1 | 39.3 | 6.2 | 111.5 | 0.710358 | 7 | - |
| Graf 10 | Rib | 102.6 | 0.5 | 45.9 | 0.8 | 89.4 | 0.709374 | 8 | - |
| Graf 11 | Longbone | 112.3 | 1.0 | 45.9 | 0.9 | 98.0 | 0.709078 | 4 | 0.000008 |
|  | Rib | 83.5 | 0.6 | 35.7 | 1.3 | 93.6 | 0.709070 | 7 |  |
| Graf 15 | Longbone | 123.7 | 4.5 | 43.2 | 1.4 | 114.6 | 0.709292 | 8 | - |
| Graf 24 | Longbone | 146.2 | 0.9 | 47.6 | 0.8 | 122.8 | 0.709746 | 8 | 0.000249 |
|  | Rib | 116.7 | 2.6 | 41.3 | 2.3 | 112.9 | 0.709497 | 6 |  |
| Graf 27 | Longbone | 143.8 | 2.6 | 40.9 | 1.0 | 140.7 | 0.709016 | 9 | - |


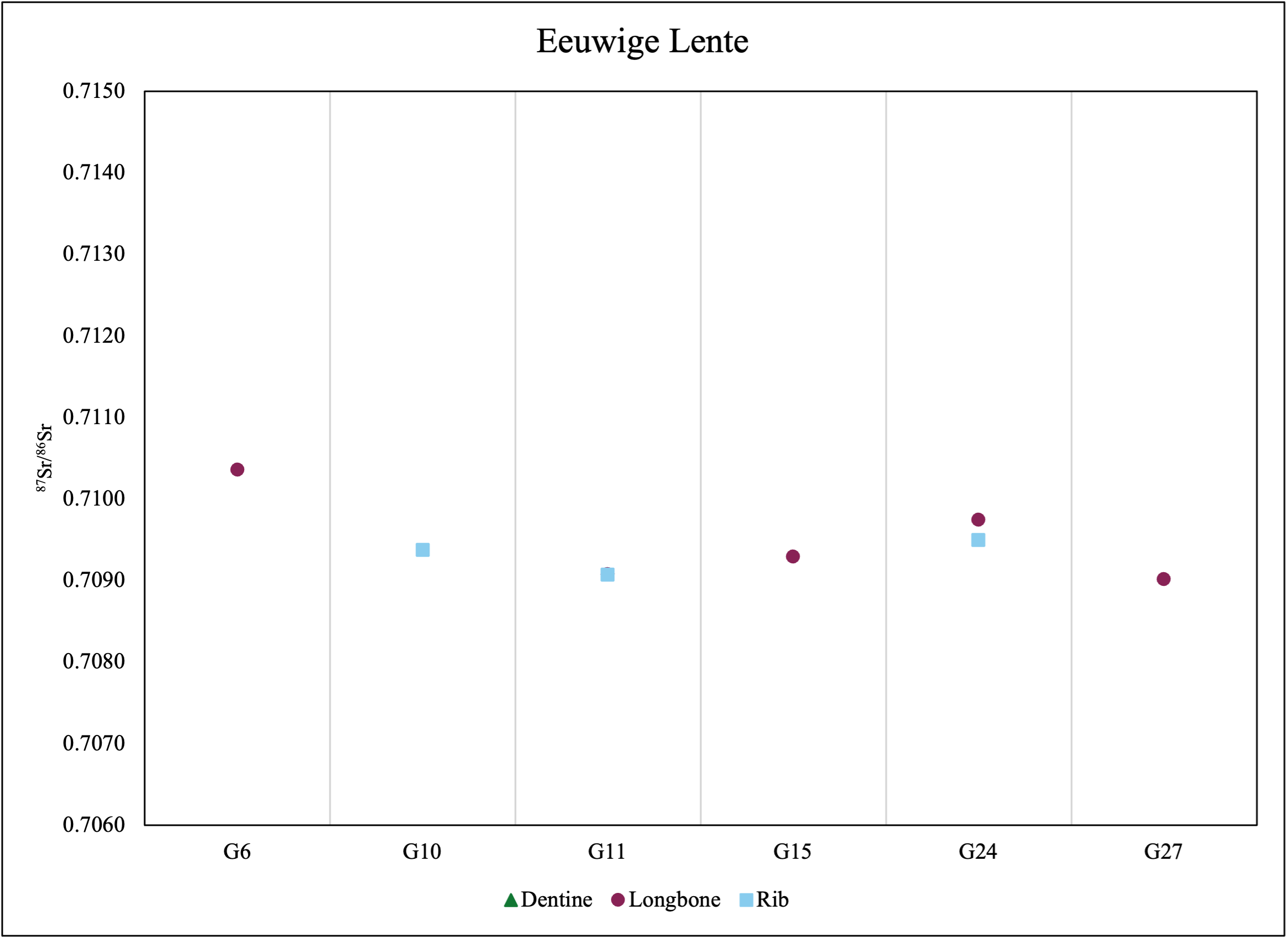


1. **Nijmegen - Laauwikstraat-Zuid (La2) [unpublished]**

| **Individual** | **Skeletal element** | **[Sr] in ppm** | **%RSD** | **[Ca] in %** | **%RSD** | **normalized [Sr]** | **^87^Sr/^86^Sr** | **2SE** | **Intra-ind. diff.** |
| --- | --- | --- | --- | --- | --- | --- | --- | --- | --- |
| Graf 2 | Dentine | 106.3 | 1.7 | 38.7 | 2.7 | 109.9 | 0.709168 | 9 | 0.000206 |
|  | Longbone | 106.0 | 5.7 | 39.7 | 1.8 | 106.8 | 0.709374 | 10 |  |
| Graf 7 | Rib | 91.6 | 2.6 | 41.9 | 4.0 | 87.5 | 0.709161 | 8 | - |
| Graf 10 | Longbone | 135.0 | 1.8 | 42.7 | 2.9 | 126.5 | 0.710034 | 6 | 0.000566 |
|  | Rib | 112.6 | 1.9 | 37.7 | 2.5 | 119.6 | 0.709468 | 7 |  |


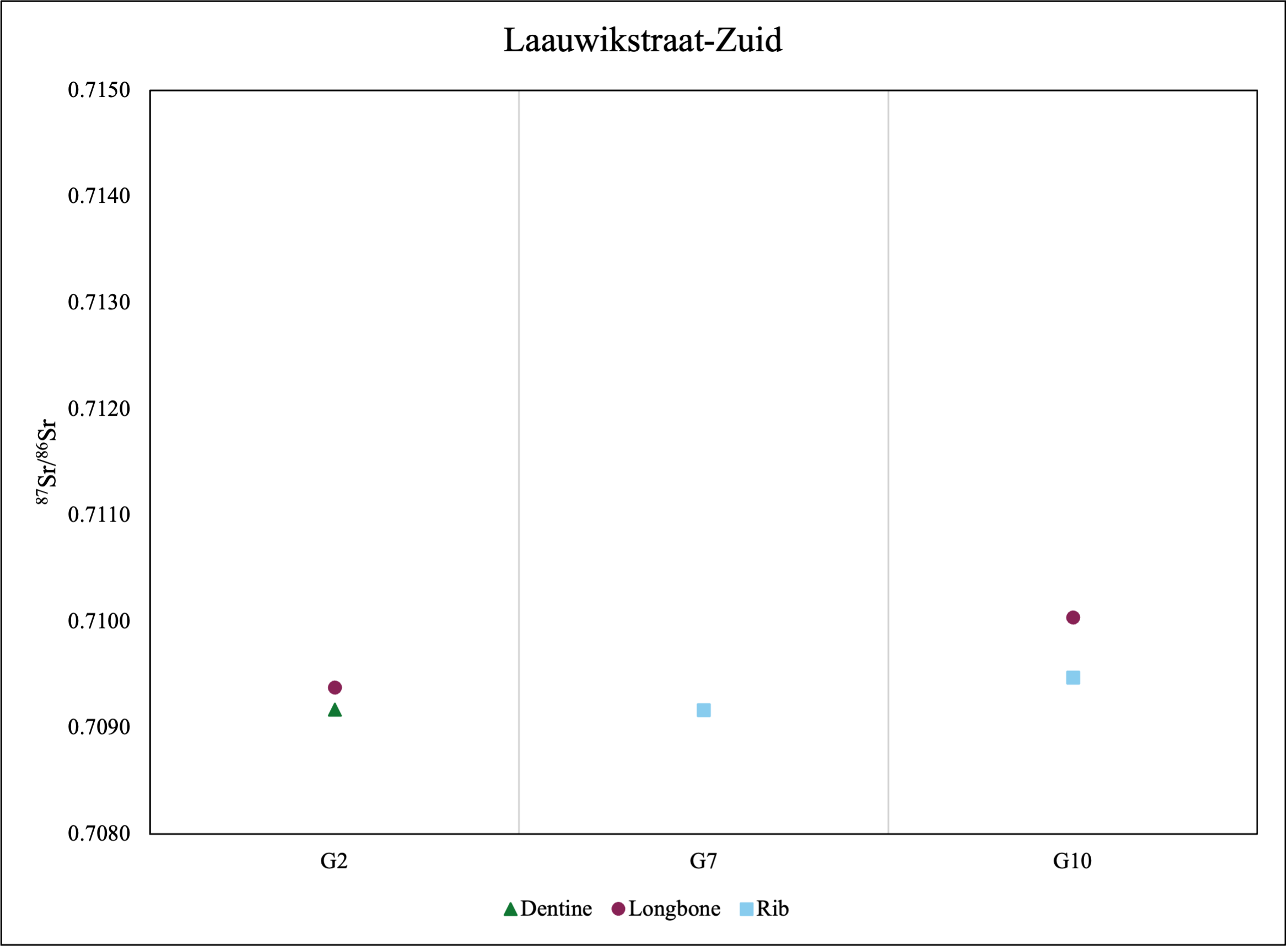


1. **Nijmegen – Lentseveld (Lv9) [2]**

| **Individual** | **Skeletal element** | **[Sr] in ppm** | **%RSD** | **[Ca] in %** | **%RSD** | **normalized [Sr]** | **^87^Sr/^86^Sr** | **2SE** | **Intra-ind. diff.** |
| --- | --- | --- | --- | --- | --- | --- | --- | --- | --- |
| Graf 3 | Dentine | 108.6 | 3.5 | 39.5 | 1.2 | 109.9 | 0.708909 | 6 | 0.000006 |
|  | Rib | 95.8 | 1.0 | 43.4 | 2.8 | 88.3 | 0.708915 | 9 |  |
| Graf 4 | Longbone | 188.9 | 2.2 | 39.0 | 2.3 | 193.7 | 0.711529 | 6 | 0.000321 |
|  | Rib | 204.6 | 2.4 | 42.3 | 3.3 | 193.5 | 0.711208 | 6 |  |
| Graf 7 | Longbone | 142.7 | 4.5 | 40.6 | 7.0 | 140.6 | 0.710984 | 8 | 0.000917 |
|  | Rib | 131.9 | 2.5 | 42.8 | 4.1 | 123.2 | 0.710067 | 7 |  |
| Graf 8 | Longbone | 104.4 | 0.8 | 39.4 | 4.2 | 105.9 | 0.709209 | 10 | - |
| Graf 11 | Longbone | 163.5 | 5.3 | 43.3 | 3.5 | 150.9 | 0.709069 | 8 | - |
| Graf 10 | Longbone | 142.5 | 3.7 | 36.9 | 3.6 | 154.4 | 0.709006 | 8 | - |
| Graf 12 | Dentine | 110.3 | 1.3 | 40.1 | 2.9 | 110.1 | 0.710006 | 8 | 0.000349 |
|  | Longbone | 114.1 | 3.5 | 42.7 | 4.2 | 106.9 | 0.709657 | 9 |  |


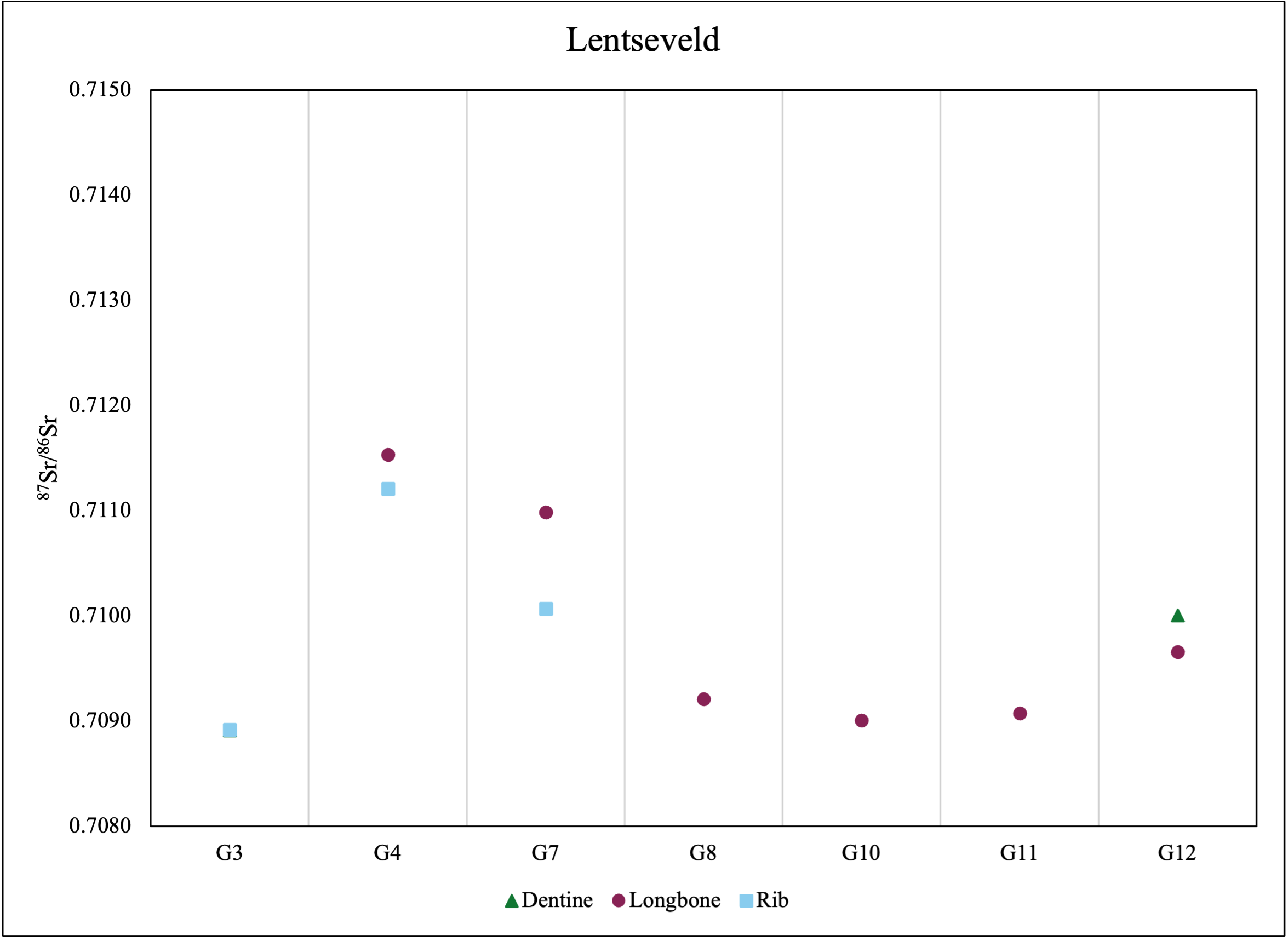


1. **Nijmegen – Steltsestraat (Sl2) [3]**

| **Individual** | **Skeletal element** | **[Sr] in ppm** | **%RSD** | **[Ca] in %** | **%RSD** | **normalized [Sr]** | **^87^Sr/^86^Sr** | **2SE** | **Intra-ind. diff.** |
| --- | --- | --- | --- | --- | --- | --- | --- | --- | --- |
| G1 | Rib | 170.5 | 2.7 | 43.9 | 2.0 | 155.5 | 0.709033 | 6 | - |
| G2 | Long bone | 107.7 | 2.8 | 41.5 | 1.8 | 103.7 | 0.709119 | 8 | - |
| G4 | Rib | 154.6 | 0.4 | 46.2 | 1.0 | 133.9 | 0.708965 | 8 | - |
| G11 | Long bone | 118.2 | 4.1 | 46.2 | 1.2 | 102.3 | 0.709322 | 6 | - |
| G26 | Long bone | 94.9 | 1.9 | 31.3 | 1.5 | 121.1 | 0.709010 | 8 | 0.000001 |
|  | Rib | 135.8 | 1.2 | 41.3 | 0.4 | 131.4 | 0.709011 | 8 |  |
| G27 | Rib | 134.0 | 5.0 | 39.6 | 3.4 | 135.4 | 0.708985 | 8 | - |
| G29 | Long bone | 147.1 | 0.7 | 48.4 | 1.7 | 121.6 | 0.709016 | 8 | - |
| G32 | Rib | 132.6 | 3.5 | 46.1 | 1.5 | 115.1 | 0.709011 | 8 | - |


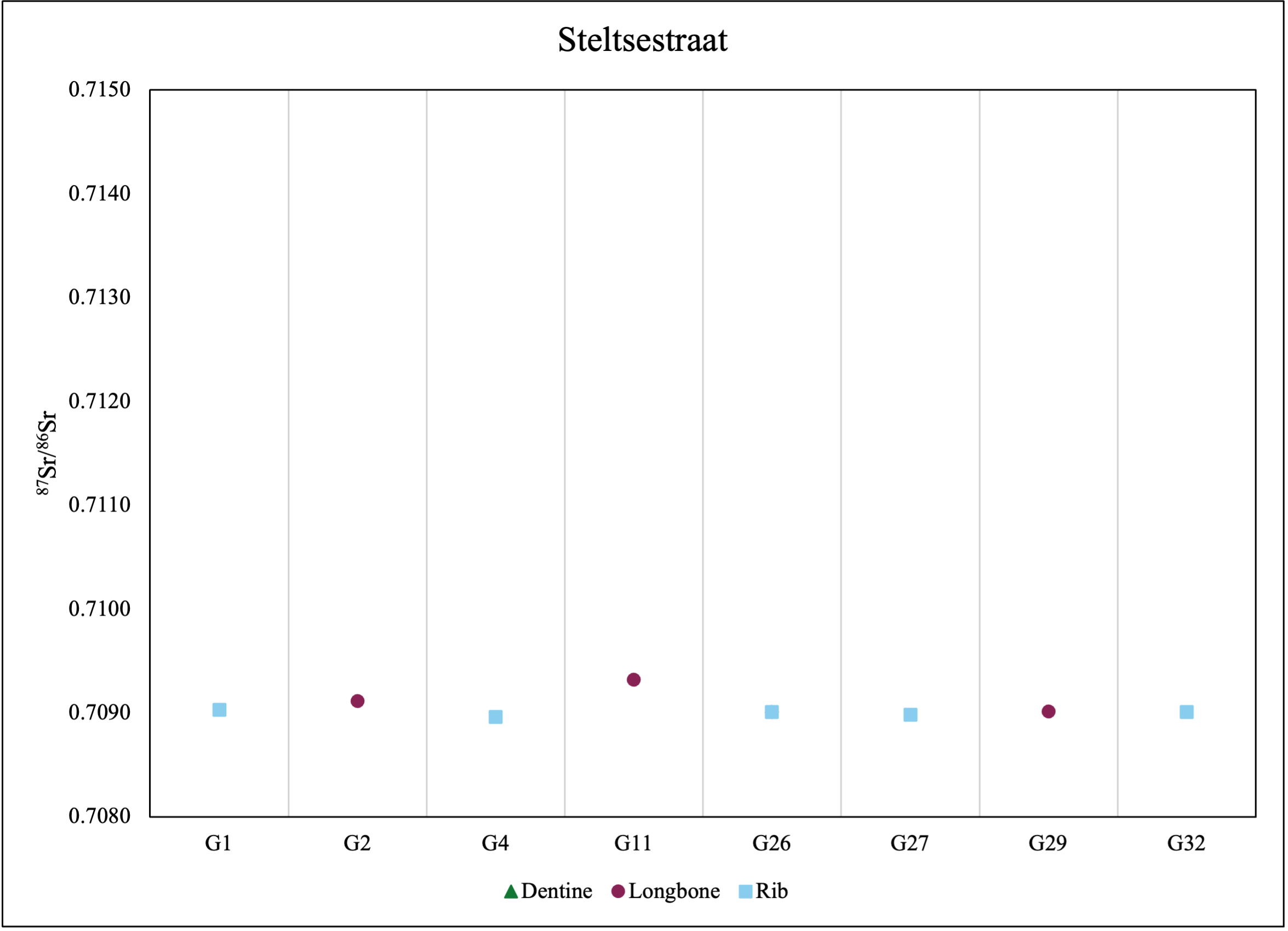


1. **Nijmegen – Zuiderveld (Zv10/Zn3) [4, 5]**

| **Individual** | **Skeletal element** | **[Sr] in ppm** | **%RSD** | **[Ca] in %** | **%RSD** | **normalized [Sr]** | **^87^Sr/^86^Sr** | **2SE** | **Intra-ind. diff.** |
| --- | --- | --- | --- | --- | --- | --- | --- | --- | --- |
| Graf 2 | Long bone | 91.6 | 3.8 | 41.7 | 5.3 | 87.7 | 0.709075 | 9 | - |
| Graf 5 | Long bone | 172.3 | 2.4 | 41.3 | 4.1 | 166.8 | 0.710538 | 8 | - |
| Graf 9 | Long bone | 76.0 | 1.7 | 41.8 | 3.1 | 72.7 | 0.709341 | 5 | 0.000236 |
|  | Rib | 84.2 | 2.7 | 41.2 | 3.2 | 81.8 | 0.709105 | 6 |  |
| Graf 10 | Long bone | 106.1 | 5.9 | 37.8 | 3.9 | 112.5 | 0.710482 | 6 | - |
| Graf 11 | Rib | 117.3 | 2.1 | 51.1 | 2.2 | 91.8 | 0.709134 | 7 | - |
| Graf 14 | Rib | 95.2 | 2.3 | 42.8 | 3.5 | 88.9 | 0.709458 | 7 | - |
| Graf 15 | Long bone | 91.7 | 3.1 | 41.5 | 2.5 | 88.5 | 0.709954 | 6 | - |
| Graf 24 | Long bone | 99.0 | 3.3 | 29.3 | 4.0 | 135.2 | 0.709086 | 7 | 0.000088 |
|  | Rib | 163.9 | 2.7 | 46.5 | 3.1 | 141.1 | 0.708998 | 8 |  |
| Graf 27 | Long bone | 286.8 | 2.1 | 91.3 | 7.2 | 125.7 | 0.709008 | 7 | 0.000043 |
|  | Rib | 132.4 | 5.0 | 43.5 | 2.9 | 121.8 | 0.708965 | 9 |  |


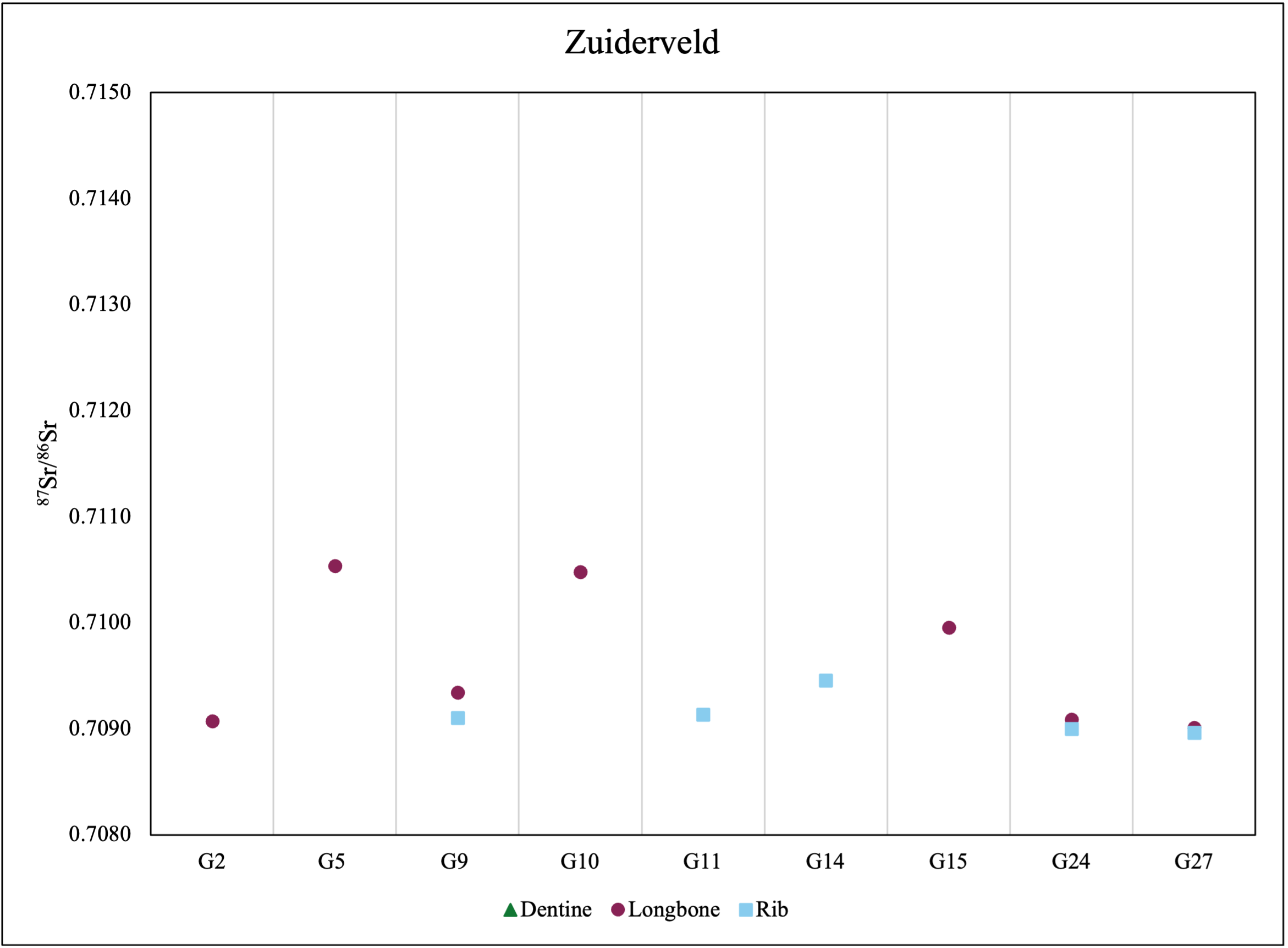


1. **Nijmegen - Trajanusplein* (ROB 1974) [6]**

| **Individual** | **Skeletal element** | **[Sr] in ppm** | **%RSD** | **[Ca] in %** | **%RSD** | **normalized [Sr]** | **^87^Sr/^86^Sr*** | **2SE** | **Intra-ind. diff.** |
| --- | --- | --- | --- | --- | --- | --- | --- | --- | --- |
| Graf 60/9 | Long bone | 101.8 | 0.6 | 42.4 | 0.3 | 96.0 | 0.710294 | 7 | 0.001829 |
|  | Rib | 190.7 | 7.5 | 52.5 | 0.4 | 145.4 | 0.712123 | 8 |  |
| Graf 60/21 | Long bone | 82.8 | 3.9 | 42.8 | 0.4 | 77.5 | 0.710524 | 8 | 0.000016 |
|  | Rib | 90.6 | 0.6 | 40.3 | 0.1 | 89.9 | 0.710540 | 7 |  |
| Graf 60/22 | Long bone | 80.5 | 0.4 | 39.5 | 0.3 | 81.5 | 0.710704 | 8 | 0.000077 |
|  | Rib | 179.4 | 1.7 | 38.9 | 0.2 | 184.5 | 0.710781 | 8 |  |

* Published in Roymans, N., van den Dikkenberg, L., & Kootker, L. (2024). Societal change and interregional connectivity in the 5th-century BC Lower Rhine-Meuse region. In *Chariots on Fire, Reins of Power: Early La Tène elite burials from the Lower Rhine-Meuse region and their Northwest European context*(pp. 481-524). Sidestone Press.

1. **Nijmegen - Kops Plateau* (ROB 1986-1995) [7]**

| **Individual** | **Skeletal element** | **[Sr] in ppm** | **%RSD** | **[Ca] in %** | **%RSD** | **normalized [Sr]** | **^87^Sr/^86^Sr*** | **2SE** | **Intra-ind. diff.** |
| --- | --- | --- | --- | --- | --- | --- | --- | --- | --- |
| G72 | Long bone | 78.9 | 0.8 | 33.6 | 0.1 | 94.0 | 0.710773 | 8 | 0.000044 |
|  | Rib | 89.2 | 0.7 | 37.2 | 0.4 | 95.9 | 0.710817 | 8 |  |
| G74 | Long bone | 110.1 | 4.9 | 48.7 | 0.2 | 90.4 | 0.710464 | 8 | - |
| G75 | Long bone | 113.5 | 0.4 | 39.2 | 0.2 | 115.8 | 0.710895 | 7 | - |
| G76 | Long bone | 221.1 | 7.7 | 106.4 | 0.4 | 83.1 | 0.710215 | 8 | 0.000216 |
|  | Rib | 248.2 | 3.9 | 105.1 | 0.5 | 94.5 | 0.709999 | 8 |  |
| G79 | Long bone | 172.2 | 0.9 | 72.5 | 0.5 | 95.0 | 0.710982 | 6 | 0.001614 |
|  | Rib | 288.0 | 8.0 | 101.1 | 0.8 | 113.9 | 0.712596 | 8 |  |

* Published in Roymans, N., van den Dikkenberg, L., & Kootker, L.M. (2024). Societal change and interregional connectivity in the 5th-century BC Lower Rhine-Meuse region. In *Chariots on Fire, Reins of Power: Early La Tène elite burials from the Lower Rhine-Meuse region and their Northwest European context*(pp. 481-524). Sidestone Press.


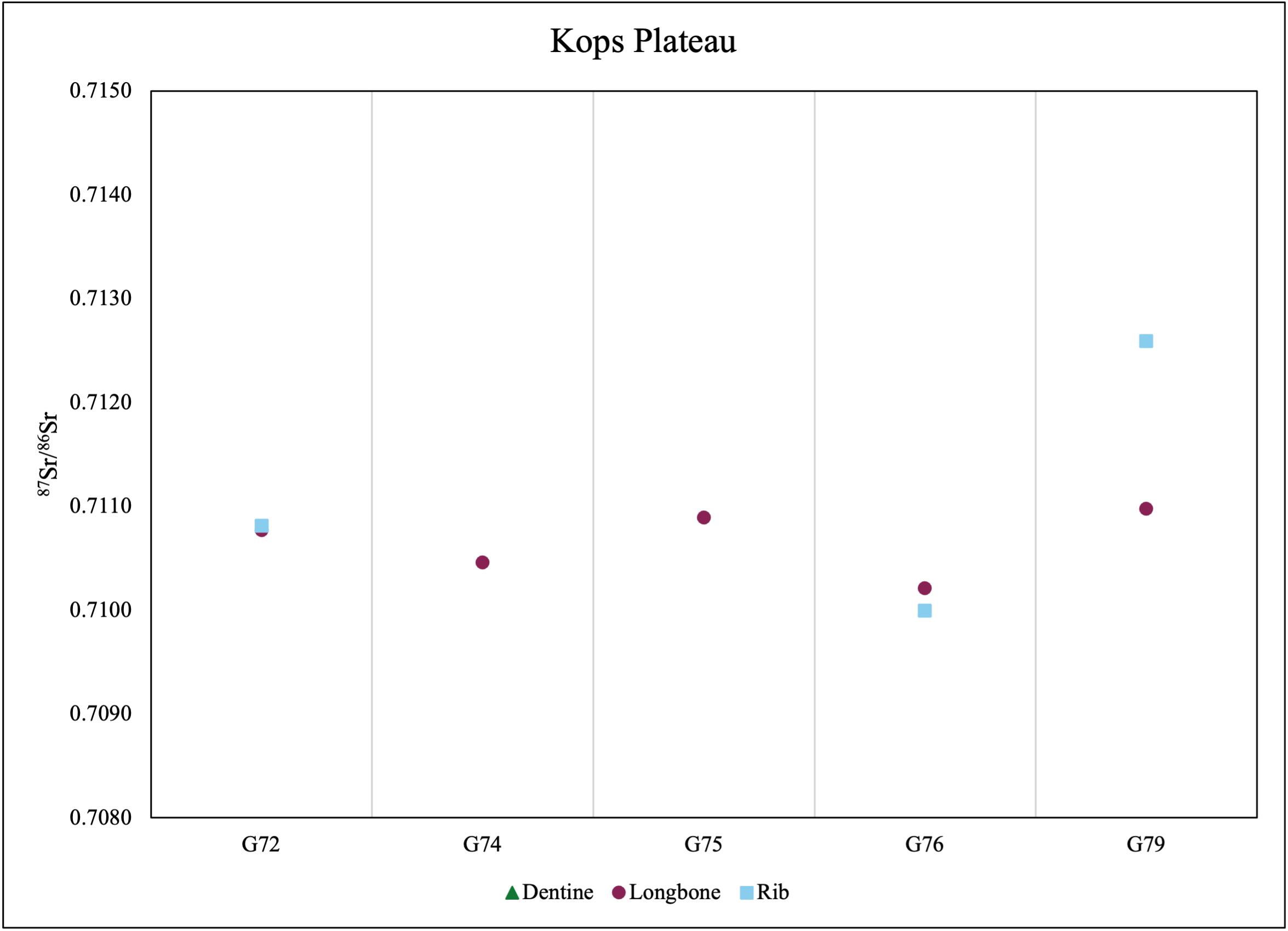


1. **Heumen* (RCE 2018) [7]**

| **Individual** | **Skeletal element** | **[Sr] in ppm** | **%RSD** | **[Ca] in %** | **%RSD** | **normalized [Sr]** | **^87^Sr/^86^Sr*** | **2SE** | **Intra-ind. diff.** |
| --- | --- | --- | --- | --- | --- | --- | --- | --- | --- |
| V182 | Long bone | 66.0 | 1.0 | 39.5 | 0.3 | 66.9 | 0.710270 | 6 | - |

* Published in Roymans, N., van den Dikkenberg, L., & Kootker, L.M. (2024). Societal change and interregional connectivity in the 5th-century BC Lower Rhine-Meuse region. In *Chariots on Fire, Reins of Power: Early La Tène elite burials from the Lower Rhine-Meuse region and their Northwest European context*(pp. 481-524). Sidestone Press.


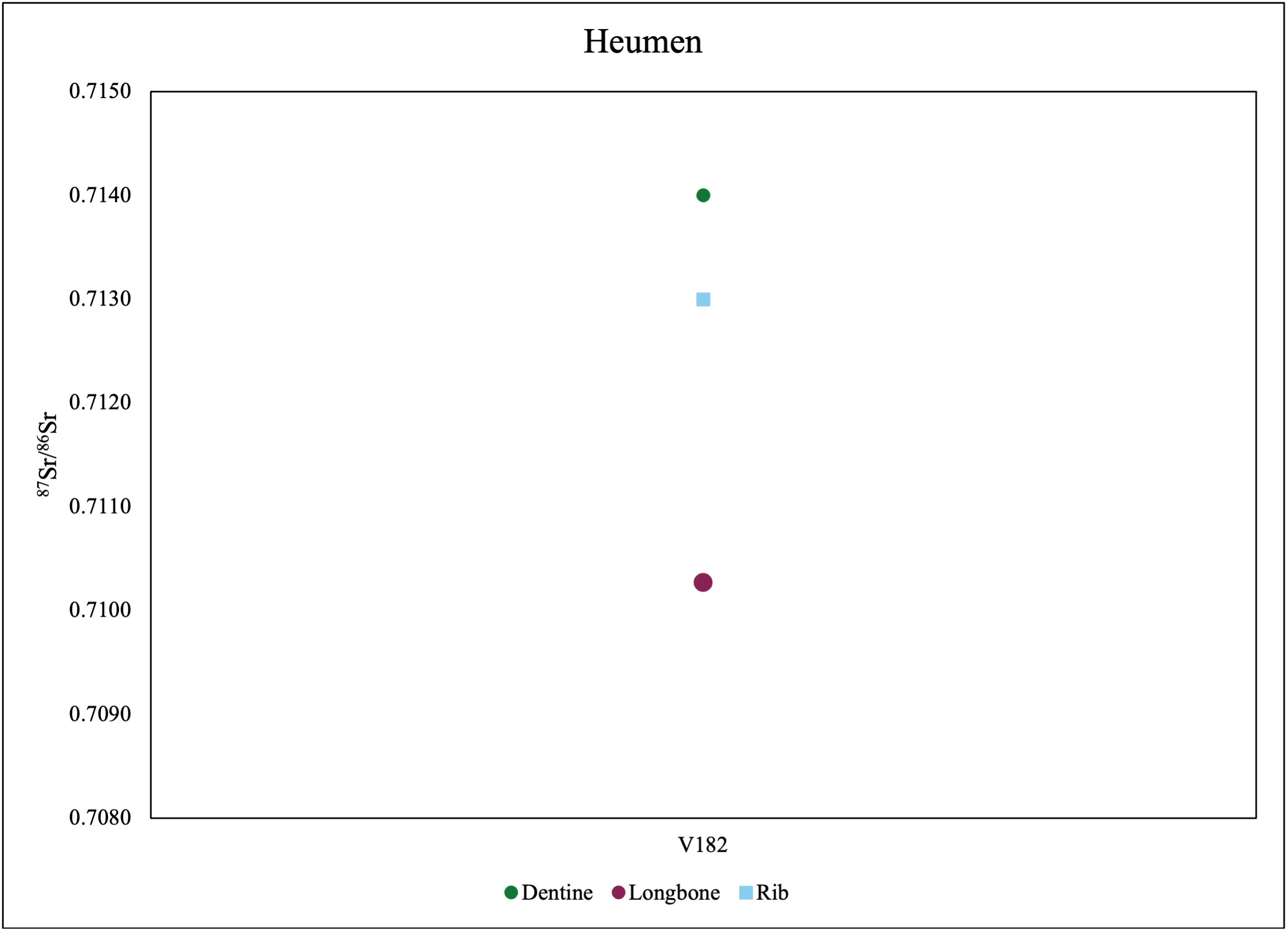


1. **Nijmegen - ’t Klumke/Griftdijk (Ngk2-9) [8]**

| **Individual** | **Skeletal element** | **[Sr] in ppm** | **%RSD** | **[Ca] in %** | **%RSD** | **normalized [Sr]** | **^87^Sr/^86^Sr** | **2SE** | **Intra-ind. diff.** |
| --- | --- | --- | --- | --- | --- | --- | --- | --- | --- |
| G1 | Long bone | 106.5 | 1.0 | 41.9 | 0.8 | 101.7 | 0.709146 | 10 | 0.000221 |
|  | Rib | - | - | - | - | - | 0.708926 | 8 |  |
| G2.1 | Long bone | 86.7 | 0.2 | 46.3 | 0.8 | 74.9 | 0.709068 | 7 | 0.000035 |
|  | Rib | 191.7 | 0.3 | 42.3 | 1.7 | 181.3 | 0.709104 | 7 |  |
| G2.2 | Long bone | 69.7 | 1.3 | 50.0 | 0.7 | 55.7 | 0.709096 | 7 | - |
| G3 | Long bone | - | - | - | - | - | 0.709387 | 8 | 0.000085 |
|  | Rib | 116.0 | 0.6 | 48.4 | 1.1 | 95.8 | 0.709472 | 7 |  |
| G4 | Long bone | 108.3 | 1.0 | 42.4 | 0.8 | 102.1 | 0.709307 | 6 | 0.000034 |
|  | Rib | 301.8 | 0.3 | 47.2 | 0.8 | 255.9 | 0.709340 | 6 |  |
| G4/II | Dentine | - | - | - | - | - | 0.709620 | 7 | - |
| G4/III | Dentine | 160.6 | 1.3 | 39.1 | 1.0 | 164.4 | 0.709429 | 7 | - |
| G5 | Long bone | - | - | - | - | - | 0.709306 | 9 | 0.000043 |
|  | Rib | - | - | - | - | - | 0.709349 | 9 |  |
| G10 | Long bone | - | - | - | - | - | 0.708968 | 7 | - |
| G15 | Long bone | 98.6 | 0.4 | 41.5 | 0.4 | 95.0 | 0.709665 | 10 | 0.000023 |
|  | Rib | 103.6 | 0.7 | 51.5 | 0.8 | 80.4 | 0.709642 | 6 |  |
| G16 | Long bone | - | - | - | - | - | 0.709353 | 9 | 0.000053 |
|  | Rib | - | - | - | - | - | 0.709300 | 8 |  |
| G17.1 | Long bone | 96.6 | 0.8 | 39.4 | 1.2 | 98.1 | 0.709264 | 8 | 0.000074 |
|  | Rib | - | - | - | - | - | 0.709338 | 7 |  |
| G17.2 | Long bone | - | - | - | - | - | 0.709390 | 7 | 0.000280 |
|  | Rib | 95.1 | 0.9 | 46.9 | 1.1 | 81.1 | 0.709111 | 7 |  |
| G19 | Long bone | 94.8 | 0.3 | 39.7 | 1.4 | 95.5 | 0.709082 | 8 | 0.000010 |
|  | Rib | - | - | - | - | - | 0.709091 | 9 |  |
| G20 | Long bone | - | - | - | - | - | 0.709322 | 7 | 0.000020 |
|  | Rib | - | - | - | - | - | 0.709342 | 12 |  |
| G21 | Long bone | 114.9 | 1.9 | 51.0 | 0.6 | 90.1 | 0.709323 | 8 | 0.000091 |
|  | Rib | - | - | - | - | - | 0.709415 | 9 |  |
| G23 | Long bone | - | - | - | - | - | 0.709133 | 9 | 0.000112 |
|  | Rib | 108.6 | 0.8 | 41.8 | 0.7 | 103.8 | 0.709245 | 7 |  |
| G24 | Long bone | 146.9 | 1.0 | 47.1 | 1.5 | 124.8 | 0.709254 | 8 | 0.000080 |
|  | Rib | 148.9 | 1.2 | 45.5 | 1.1 | 130.8 | 0.709334 | 8 |  |
| G25 | Long bone | 96.4 | 0.9 | 46.0 | 0.8 | 83.9 | 0.709315 | 8 | - |
| G26 | Long bone | - | - | - | - | - | 0.709135 | 8 | 0.002940 |
|  | Rib | 296.8 | 0.9 | 76.1 | 0.5 | 156.1 | 0.712075 | 9 |  |
| G27 | Long bone | 218.1 | 1.0 | 39.7 | 1.5 | 219.6 | 0.709238 | 8 | 0.000026 |
|  | Rib | - | - | - | - | - | 0.709263 | 7 |  |
| G28 | Long bone | 90.7 | 0.8 | 40.7 | 1.0 | 89.3 | 0.709170 | 8 | 0.000124 |
|  | Rib | 120.4 | 0.2 | 44.9 | 1.0 | 107.2 | 0.709046 | 7 |  |
| 168 | Dentine | 135.6 | 1.4 | 43.8 | 0.7 | 123.9 | 0.709210 | 7 | 0.000379  0.000435 |
|  | Long bone | 272.0 | 1.0 | 41.9 | 1.9 | 259.7 | 0.709589 | 8 |  |
|  | Rib | 96.1 | 0.3 | 37.8 | 0.6 | 101.7 | 0.709154 | 8 |  |
| 198 | Long bone | 110.1 | 0.6 | 42.8 | 0.4 | 102.9 | 0.709367 | 10 | 0.000043 |
|  | Rib | 117.1 | 0.7 | 43.6 | 2.3 | 107.5 | 0.709323 | 7 |  |
| 237 | Long bone | 149.0 | 1.3 | 35.1 | 0.6 | 169.7 | 0.709341 | 7 | 0.000004 |
|  | Rib | 109.1 | 0.9 | 41.5 | 1.4 | 105.2 | 0.709345 | 9 |  |
| 263 | Long bone | 124.8 | 1.5 | 48.1 | 1.2 | 103.8 | 0.709291 | 7 | 0.000048 |
|  | Rib | 144.9 | 0.8 | 42.4 | 0.8 | 136.8 | 0.709339 | 8 |  |
| 273 | Long bone | 169.2 | 2.4 | 45.3 | 0.8 | 149.5 | 0.709854 | 7 | 0.000532 |
|  | Rib | - | - | - | - | - | 0.710386 | 7 |  |
| 2280 | Dentine | 146.2 | 1.0 | 45.1 | 0.6 | 129.6 | 0.709943 | 7 | 0.000273  0.000038 |
|  | Long bone | 122.8 | 0.2 | 40.6 | 0.5 | 120.9 | 0.709670 | 7 |  |
|  | Rib | 180.7 | 0.6 | 43.6 | 0.2 | 165.8 | 0.709632 | 8 |  |


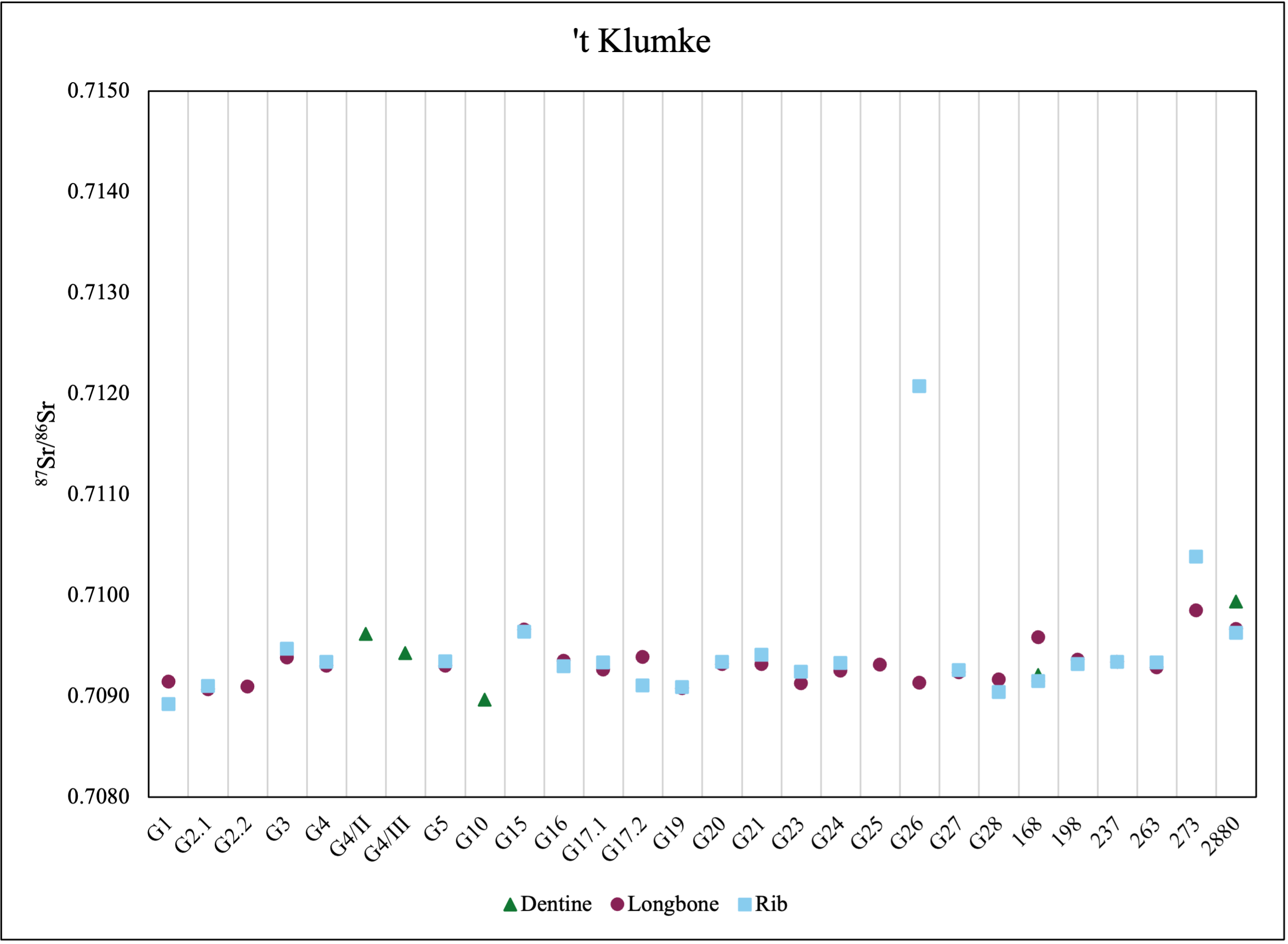


1. **Nijmegen - Rust Wat (Bo6/8) [9]**

| **Individual** | **Skeletal element** | **[Sr] in ppm** | **%RSD** | **[Ca] in %** | **%RSD** | **normalized [Sr]** | **^87^Sr/^86^Sr** | **2SE** | **Intra-ind. diff.** |
| --- | --- | --- | --- | --- | --- | --- | --- | --- | --- |
| G9 | Long bone | 839.2 | 0.1 | 241.5 | 1.8 | 139.0 | 0.709199 | 10 | - |
| G10 | Long bone | - | - | - | - | - | 0.709191 | 6 | 0.000012 |
|  | Rib | - | - | - | - | - | 0.709203 | 9 |  |
| G11 | Rib | 109.6 | 1.4 | 60.9 | 0.6 | 72.0 | 0.710535 | 8 | - |
| G12 | Long bone | 174.4 | 0.4 | 48.8 | 0.9 | 143.1 | 0.709252 | 8 | 0.000028 |
|  | Rib | 125.5 | 0.6 | 46.1 | 1.6 | 108.8 | 0.709280 | 7 |  |
| G15 | Long bone | - | - | - | - | - | 0.709129 | 8 | - |
| G17 | Long bone | 85.9 | 1.8 | 32.0 | 1.2 | 107.2 | 0.709141 | 6 | - |
| G18 | Long bone | - | - | - | - | - | 0.709691 | 8 | - |
| G22 | Long bone | - | - | - | - | - | 0.709490 | 8 | 0.000249 |
|  | Rib | - | - | - | - | - | 0.709739 | 9 |  |
| G40 | Rib | - | - | - | - | - | 0.709272 | 8 | - |
| G42 | Long bone | - | - | - | - | - | 0.709590 | 9 | 0.000024 |
|  | Rib | - | - | - | - | - | 0.709614 | 8 |  |
| G45 | Long bone | - | - | - | - | - | 0.709280 | 8 | 0.000014 |
|  | Rib | - | - | - | - | - | 0.709294 | 9 |  |
| G50 | Long bone | - | - | - | - | - | 0.709380 | 9 | 0.000072 |
|  | Rib | - | - | - | - | - | 0.709308 | 10 |  |
| G56 | Long bone | - | - | - | - | - | 0.709747 | 6 | 0.000365 |
|  | Rib | - | - | - | - | - | 0.709382 | 8 |  |
| G58 | Long bone | - | - | - | - | - | 0.709310 | 7 | 0.000061 |
|  | Rib | - | - | - | - | - | 0.709249 | 6 |  |
| G63 | Long bone | - | - | - | - | - | 0.709531 | 8 | - |
| G64 | Long bone | - | - | - | - | - | 0.709344 | 6 | 0.000293 |
|  | Rib | - | - | - | - | - | 0.709637 | 6 |  |
| G65 | Long bone | - | - | - | - | - | 0.709210 | 6 | 0.000021 |
|  | Rib | - | - | - | - | - | 0.709189 | 8 |  |
| G66 | Long bone | - | - | - | - | - | 0.709150 | 8 | 0.000019 |
|  | Rib | 120.0 | 1.4 | 49.2 | 0.9 | 97.6 | 0.709131 | 8 |  |
| G68 | Long bone | 147.2 | 0.8 | 64.9 | 2.5 | 90.8 | 0.709385 | 7 | 0.000115 |
|  | Rib | - | - | - | - | - | 0.709270 | 7 |  |
| G72 | Long bone | - | - | - | - | - | 0.709579 | 8 | 0.000133 |
|  | Rib | - | - | - | - | - | 0.709712 | 8 |  |
| G75 | Long bone | - | - | - | - | - | 0.709477 | 8 | - |
| G76 | Long bone | - | - | - | - | - | 0.709277 | 7 | 0.000037 |
|  | Rib | 112.6 | 0.4 | 48.7 | 0.5 | 92.4 | 0.709314 | 8 |  |
| G77 | Rib | - | - | - | - | - | 0.709332 | 12 | - |
| G82 | Long bone | - | - | - | - | - | 0.709915 | 7 | 0.000515 |
|  | Rib | 113.6 | 1.2 | 46.1 | 1.4 | 98.6 | 0.709400 | 10 |  |
| G84 | Long bone | 140.3 | 1.7 | 41.6 | 0.4 | 134.8 | 0.709198 | 7 | 0.000046 |
|  | Rib | 117.4 | 1.0 | 44.8 | 0.6 | 104.9 | 0.709152 | 7 |  |
| G87 | Long bone | 152.0 | 0.7 | 45.3 | 0.4 | 134.3 | 0.709159 | 6 | 0.000569 |
|  | Rib | 79.4 | 0.6 | 45.7 | 0.6 | 69.5 | 0.709728 | 7 |  |
| G88 | Long bone | - | - | - | - | - | 0.709415 | 8 | - |
| G92 | Long bone | 159.5 | 1.0 | 50.6 | 0.9 | 126.2 | 0.709544 | 8 | 0.000231 |
|  | Rib | - | - | - | - | - | 0.709775 | 8 |  |


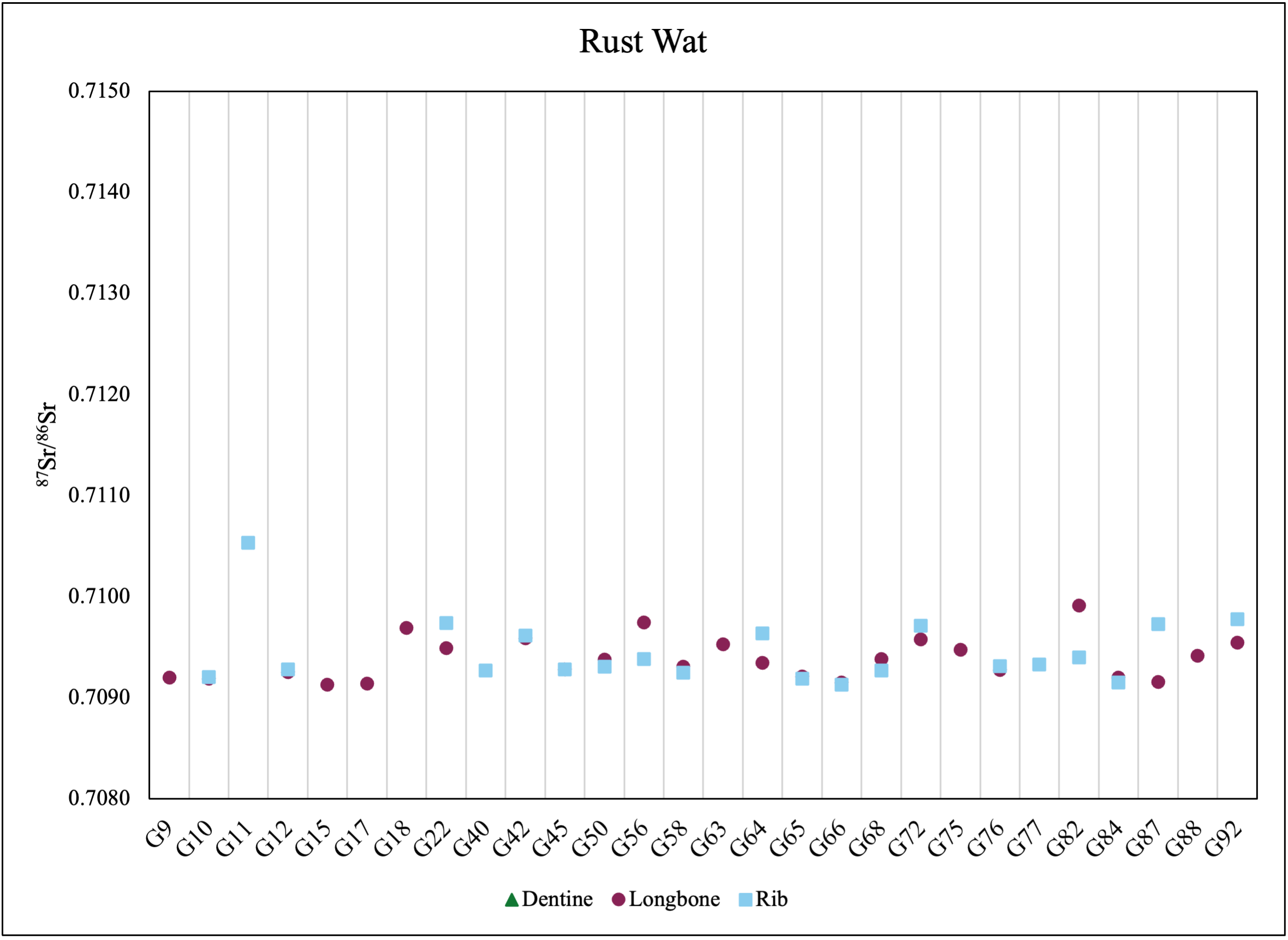


1. **Nijmegen – Broodkorf (Nlz9/13_Brk1) [10]**

| **Individual** | **Skeletal element** | **[Sr] in ppm** | **%RSD** | **[Ca] in %** | **%RSD** | **normalized [Sr]** | **^87^Sr/^86^Sr** | **2SE** | **Intra-ind. diff.** |
| --- | --- | --- | --- | --- | --- | --- | --- | --- | --- |
| V15 | Long bone | 90.2 | 0.3 | 41.0 | 1.6 | 88.0 | 0.709405 | 8 | - |
| V45 | Rib | 160.9 | 1.0 | 41.4 | 1.6 | 155.3 | 0.709503 | 8 | - |
| V390 | Dentine | 99.3 | 1.5 | 41.6 | 0.3 | 95.5 | 0.709335 | 8 | 0.000116 |
|  | Long bone | 135.5 | 1.1 | 37.1 | 1.2 | 146.1 | 0.709219 | 8 |  |
| V460 | Long bone | 102.0 | 0.5 | 40.5 | 1.0 | 100.8 | 0.709386 | 7 | - |
| V750 | Long bone | - | - | - | - | - | 0.709247 | 8 |  |
|  | Rib | 182.3 | 0.9 | 38.4 | 0.4 | 189.8 | 0.709314 | 8 | 0.000067 |


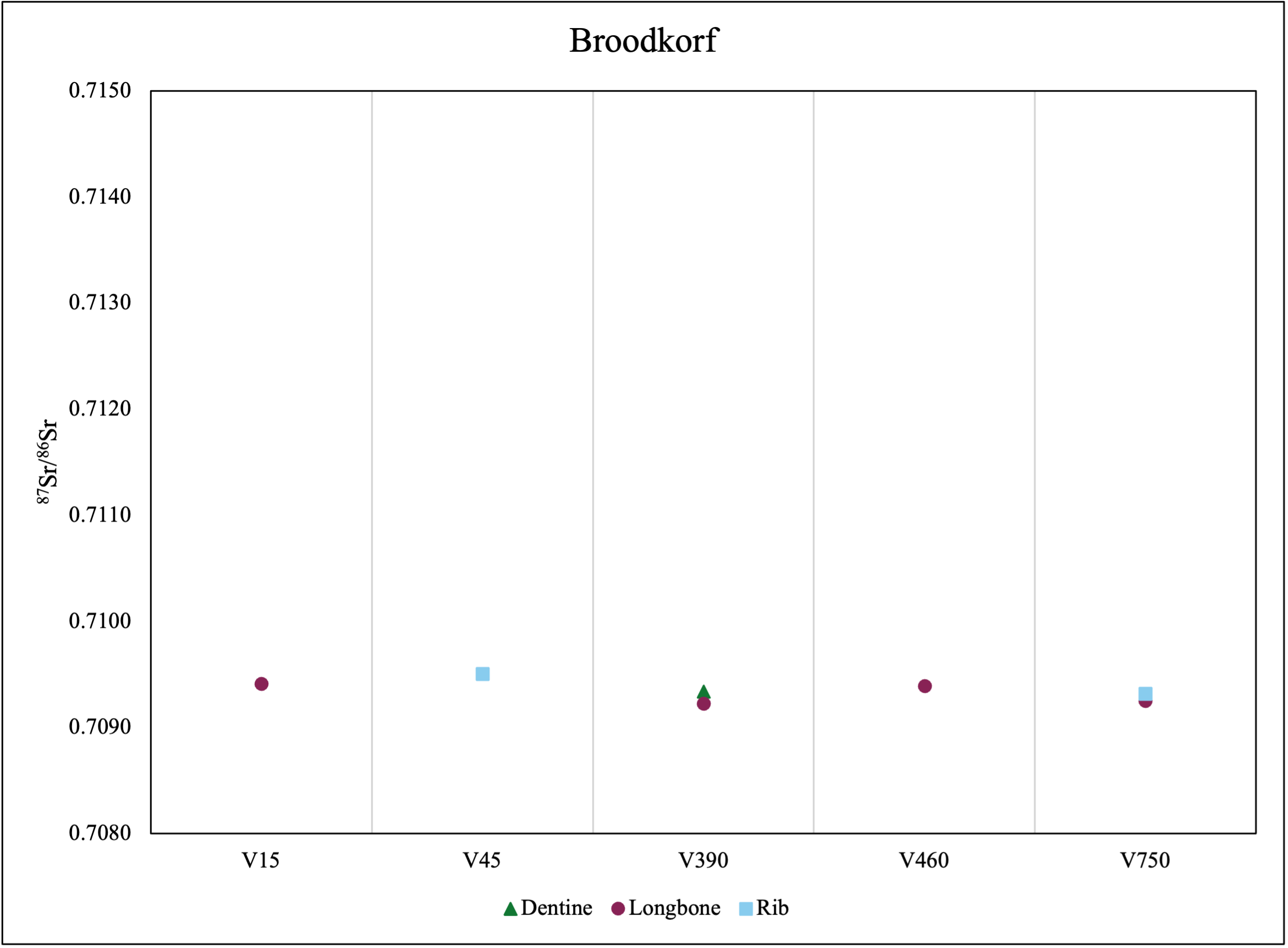


1. **Nijmegen - Onder Hees (Vr8/KUN) [11]**

| **Individual** | **Skeletal element** | **[Sr] in ppm** | **%RSD** | **[Ca] in %** | **%RSD** | **normalized [Sr]** | **^87^Sr/^86^Sr** | **2SE** | **Intra-**  **ind. diff.** |
| --- | --- | --- | --- | --- | --- | --- | --- | --- | --- |
| 226 | Long bone | 179.8 | 1.3 | 46.9 | 0.5 | 153.3 | 0.710131 | 9 | 0.000610 |
|  | Rib | 219.6 | 1.2 | 55.0 | 1.2 | 159.8 | 0.709995 | 10 |  |
| 282 | Long bone | 112.9 | 0.2 | 43.6 | 1.5 | 103.5 | 0.710115 | 8 | 0.000406 |
|  | Rib | 140.8 | 2.3 | 44.2 | 0.9 | 127.3 | 0.709709 | 7 |  |
| 295 | Dentine | - | - | - | - | - | 0.709213 | 9 | 0.000318 |
|  | Rib | 109.6 | 0.7 | 44.1 | 0.4 | 99.5 | 0.709531 | 8 |  |
| 299 | Long bone | - | - | - | - | - | 0.709489 | 11 | - |
| 303 | Long bone | 253.3 | 0.8 | 57.6 | 1.3 | 176.0 | 0.711338 | 9 | 0.000154 |
|  | Rib | 223.4 | 0.9 | 66.3 | 1.3 | 134.8 | 0.711184 | 7 |  |
| 311+312 | Long bone | - | - | - | - | - | 0.709835 | 7 | 0.000030 |
|  | Rib | 156.8 | 1.1 | 46.9 | 0.8 | 133.8 | 0.709805 | 9 |  |
| 312 | Long bone | 140.7 | 0.5 | 33.8 | 0.8 | 166.4 | 0.710050 | 8 | 0.000113 |
|  | Rib | 203.5 | 0.2 | 54.8 | 0.9 | 148.7 | 0.709937 | 8 |  |
| 315 | Long bone | 229.2 | 1.1 | 56.0 | 1.1 | 163.8 | 0.709938 | 8 | 0.000051 |
|  | Rib | 188.1 | 0.4 | 44.9 | 0.5 | 167.6 | 0.709887 | 8 |  |
| 316 | Long bone | - | - | - | - | - | 0.708804 | 8 | - |
| 318 | Long bone | 145.4 | 0.7 | 56.1 | 1.0 | 103.8 | 0.710466 | 7 | - |
| 319b | Long bone | - | - | - | - | - | 0.709716 | 7 | 0.000065 |
|  | Rib | 201.5 | 0.5 | 65.4 | 0.7 | 123.2 | 0.709781 | 7 |  |
| 320 | Long bone | 319.6 | 0.2 | 60.2 | 0.4 | 212.4 | 0.709667 | 9 | 0.000027 |
|  | Rib | - | - | - | - | - | 0.709694 | 7 |  |
| 335 | Long bone | 110.4 | 1.1 | 27.7 | 0.7 | 159.4 | 0.709348 | 8 | 0.000013 |
|  | Rib | 396.3 | 1.0 | 80.7 | 0.3 | 196.5 | 0.709361 | 6 |  |
| 336 | Long bone | 359.8 | 1.1 | 54.5 | 0.5 | 264.1 | 0.709961 | 8 | 0.000537 |
|  | Rib | 170.4 | 0.3 | 47.9 | 0.0 | 142.3 | 0.710498 | 8 |  |
| 340 | Long bone | - | - | - | - | - | 0.709791 | 9 | - |
| 341 | Long bone | 98.7 | 0.7 | 25.9 | 1.4 | 152.3 | 0.709479 | 7 | - |
| 342 | Dentine | 163.0 | 0.6 | 47.1 | 1.6 | 138.3 | 0.709185 | 7 | 0.000117  0.000432 |
|  | Long bone | 180.5 | 1.1 | 39.1 | 1.5 | 184.7 | 0.709068 | 9 |  |
|  | Rib | 190.6 | 1.6 | 46.5 | 0.2 | 164.0 | 0.709500 | 8 |  |
| 343 | Long bone | - | - | - | - | - | 0.709739 | 7 | - |
| 348 | Rib | 260.0 | 1.5 | 59.6 | 0.6 | 174.5 | 0.709556 | 6 | - |
| 351 | Dentine | - | - | - | - | - | 0.709453 | 8 | 0.000109  0.000034 |
|  | Long bone | 263.2 | 0.6 | 65.5 | 0.1 | 160.7 | 0.709562 | 9 |  |
|  | Rib | - | - | - | - | - | 0.709528 | 8 |  |
| 354 | Dentine | 129.8 | 0.7 | 38.4 | 0.5 | 135.4 | 0.709788 | 8 | 0.000103 |
|  | Rib | 164.0 | 1.0 | 28.4 | 0.3 | 231.3 | 0.709685 | 8 |  |
| 357 | Long bone | - | - | - | - | - | 0.709867 | 12 | 0.000051 |
|  | Rib | - | - | - | - | - | 0.709918 | 7 |  |
| 359 | Dentine | 148.6 | 0.4 | 44.6 | 0.5 | 133.2 | 0.709701 | 8 | 0.000248  0.000222 |
|  | Long bone | - | - | - | - | - | 0.709949 | 8 |  |
|  | Rib | 356.9 | 0.7 | 41.9 | 0.5 | 340.4 | 0.710171 | 6 |  |
| 363 | Long bone | 303.8 | 1.1 | 68.6 | 0.2 | 177.2 | 0.709849 | 3 | 0.000303 |
|  | Rib | 212.9 | 0.9 | 51.5 | 0.6 | 165.5 | 0.709546 | 8 |  |
| 364 | Long bone | 338.5 | 1.1 | 68.8 | 0.3 | 196.9 | 0.709747 | 8 | 0.000189 |
|  | Rib | 301.0 | 0.8 | 66.9 | 0.3 | 179.9 | 0.709558 | 8 |  |
| 367 | Long bone | 232.7 | 1.1 | 50.4 | 1.6 | 184.8 | 0.709720 | 7 | 0.000005 |
|  | Rib | 149.5 | 1.1 | 62.5 | 1.1 | 95.6 | 0.709715 | 8 |  |
| 369 | Rib | 190.5 | 0.4 | 66.6 | 0.6 | 114.4 | 0.709772 | 8 | - |
| 370 | Long bone | 299.2 | 1.4 | 62.2 | 1.0 | 192.5 | 0.709749 | 7 | 0.000024 |
|  | Rib | - | - | - | - | - | 0.709773 | 8 |  |
| 382 | Long bone | 193.1 | 1.0 | 50.1 | 0.7 | 154.3 | 0.709560 | 7 | 0.000173 |
|  | Rib | 239.5 | 0.7 | 65.4 | 0.8 | 146.4 | 0.709733 | 9 |  |
| 383 | Long bone | 132.9 | 0.3 | 45.5 | 2.0 | 116.8 | 0.709676 | 7 | - |
| 384 | Long bone | 266.5 | 0.3 | 62.6 | 0.5 | 170.2 | 0.709430 | 8 | - |
| 385 | Long bone | 260.9 | 0.7 | 49.9 | 1.0 | 209.2 | 0.710000 | 8 | 0.000032 |
|  | Rib | 127.3 | 0.4 | 42.6 | 0.5 | 119.7 | 0.710032 | 8 |  |
| 386 | Long bone | 196.2 | 0.8 | 45.5 | 1.0 | 172.3 | 0.709524 | 9 | 0.000015 |
|  | Rib | 98.5 | 0.2 | 26.4 | 0.4 | 149.1 | 0.709509 | 8 |  |
| 388 | Long bone | 297.0 | 1.2 | 66.5 | 0.2 | 178.6 | 0.709109 | 7 | 0.000122 |
|  | Rib | 142.8 | 0.8 | 40.7 | 0.9 | 140.2 | 0.709231 | 8 |  |
| 390 | Long bone | - | - | - | - | - | 0.708895 | 8 | 0.000069 |
|  | Rib | 154.8 | 0.4 | 40.6 | 1.0 | 152.6 | 0.708964 | 7 |  |
| 391 | Long bone | 222.1 | 1.4 | 64.2 | 0.9 | 138.3 | 0.709873 | 8 | 0.000590 |
|  | Rib | 258.5 | 0.6 | 65.2 | 1.5 | 158.7 | 0.710463 | 8 |  |
| 393 | Long bone | 153.6 | 1.4 | 43.2 | 0.5 | 142.2 | 0.709674 | 7 | 0.000055 |
|  | Rib | 218.0 | 0.9 | 53.6 | 0.5 | 162.6 | 0.709729 | 7 |  |
| 395 | Dentine | 170.8 | 1.4 | 61.7 | 0.6 | 110.7 | 0.709893 | 8 | 0.000347  0.000032 |
|  | Long bone | 196.9 | 0.0 | 49.4 | 1.5 | 159.3 | 0.709546 | 8 |  |
|  | Rib | 214.1 | 0.5 | 60.0 | 1.2 | 142.8 | 0.709514 | 8 |  |
| 400 | Long bone | 216.2 | 0.8 | 62.4 | 0.3 | 138.6 | 0.710007 | 8 | 0.000080 |
|  | Rib | 193.2 | 0.8 | 43.8 | 0.8 | 176.5 | 0.710087 | 7 |  |
| 405 | Long bone | - | - | - | - | - | 0.710304 | 6 | - |
| 411 | Long bone | - | - | - | - | - | 0.709549 | 7 | 0.000018 |
|  | Rib | 131.0 | 1.1 | 45.3 | 0.7 | 115.7 | 0.709531 | 7 |  |
| 412 | Long bone | - | - | - | - | - | 0.708302 | 7 | - |
| 414 | Dentine | 125.1 | 0.3 | 38.5 | 1.1 | 130.0 | 0.710641 | 7 | 0.000539  0.000645 |
|  | Long bone | 176.7 | 0.2 | 41.4 | 0.8 | 170.8 | 0.711180 | 8 |  |
|  | Rib | - | - | - | - | - | 0.710535 | 7 |  |
| 415 | Dentine | 140.5 | 0.8 | 43.6 | 1.8 | 129.0 | 0.709696 | 8 | 0.000229 |
|  | Long bone | 128.7 | 1.1 | 26.3 | 0.7 | 195.4 | 0.709467 | 10 |  |
| 422 | Dentine | 140.3 | 0.3 | 46.3 | 1.0 | 121.2 | 0.709377 | 7 | 0.000093  0.000043 |
|  | Long bone | 176.6 | 0.9 | 53.7 | 1.4 | 131.5 | 0.709284 | 7 |  |
|  | Rib | 159.1 | 0.6 | 45.9 | 0.3 | 138.8 | 0.709327 | 7 |  |
| 428 | Long bone | 146.3 | 1.0 | 35.5 | 1.0 | 165.0 | 0.709827 | 8 | 0.000009 |
|  | Rib | 194.3 | 0.6 | 46.0 | 0.4 | 168.9 | 0.709836 | 7 |  |
| 430 | Long bone | 186.2 | 1.9 | 42.4 | 1.8 | 175.8 | 0.710234 | 8 | 0.000208 |
|  | Rib | - | - | - | - | - | 0.710026 | 8 |  |
| 437 | Dentine | - | - | - | - | - | 0.709924 | 9 | - |
| 438 | Long bone | 111.1 | 1.0 | 26.7 | 0.5 | 166.2 | 0.709623 | 9 | 0.000189 |
|  | Rib | 232.6 | 0.6 | 41.8 | 0.5 | 222.4 | 0.709812 | 8 |  |
| 441 | Long bone | 72.1 | 1.6 | 19.7 | 0.7 | 146.4 | 0.710131 | 7 | 0.000604 |
|  | Rib | 53.0 | 1.8 | 19.6 | 0.8 | 108.3 | 0.709527 | 6 |  |
| 460 | Dentine | 153.8 | 1.3 | 52.7 | 0.6 | 116.7 | 0.709496 | 8 | 0.000553 |
|  | Long bone | 139.4 | 0.7 | 40.8 | 1.0 | 136.8 | 0.709479 | 8 |  |
|  | Rib | - | - | - | - | - | 0.710032 | 7 |  |
| 470 | Dentine | - | - | - | - | - | 0.709671 | 7 | 0.000026 |
|  | Rib | - | - | - | - | - | 0.709645 | 7 |  |
| 471 | Long bone | 180.4 | 0.4 | 39.3 | 1.5 | 183.8 | 0.709212 | 8 | - |
| 480 | Rib | - | - | - | - | - | 0.710031 | 8 | - |
| 494 | Long bone | - | - | - | - | - | 0.709795 | 8 | 0.000000 |
|  | Rib | - | - | - | - | - | 0.709795 | 9 |  |
| 568 | Long bone | 95.3 | 0.5 | 36.4 | 1.1 | 104.6 | 0.709644 | 7 | - |
| 571 | Dentine | 143.9 | 0.6 | 43.4 | 1.4 | 132.8 | 0.709639 | 7 | -0.000032 |
|  | Rib | 193.8 | 1.1 | 45.4 | 0.7 | 170.8 | 0.709671 | 8 |  |
| 818 | Rib | 371.8 | 0.7 | 70.3 | 0.5 | 211.5 | 0.710288 | 7 | - |
| G30 | Long bone | - | - | - | - | - | 0.709894 | 7 | - |
| G8 | Dentine | - | - | - | - | - | 0.709797 | 8 | 0.000109  0.000048 |
|  | Long bone | - | - | - | - | - | 0.709688 | 8 |  |
|  | Rib | 137.9 | 1.9 | 45.6 | 0.5 | 121.0 | 0.709640 | 8 |  |


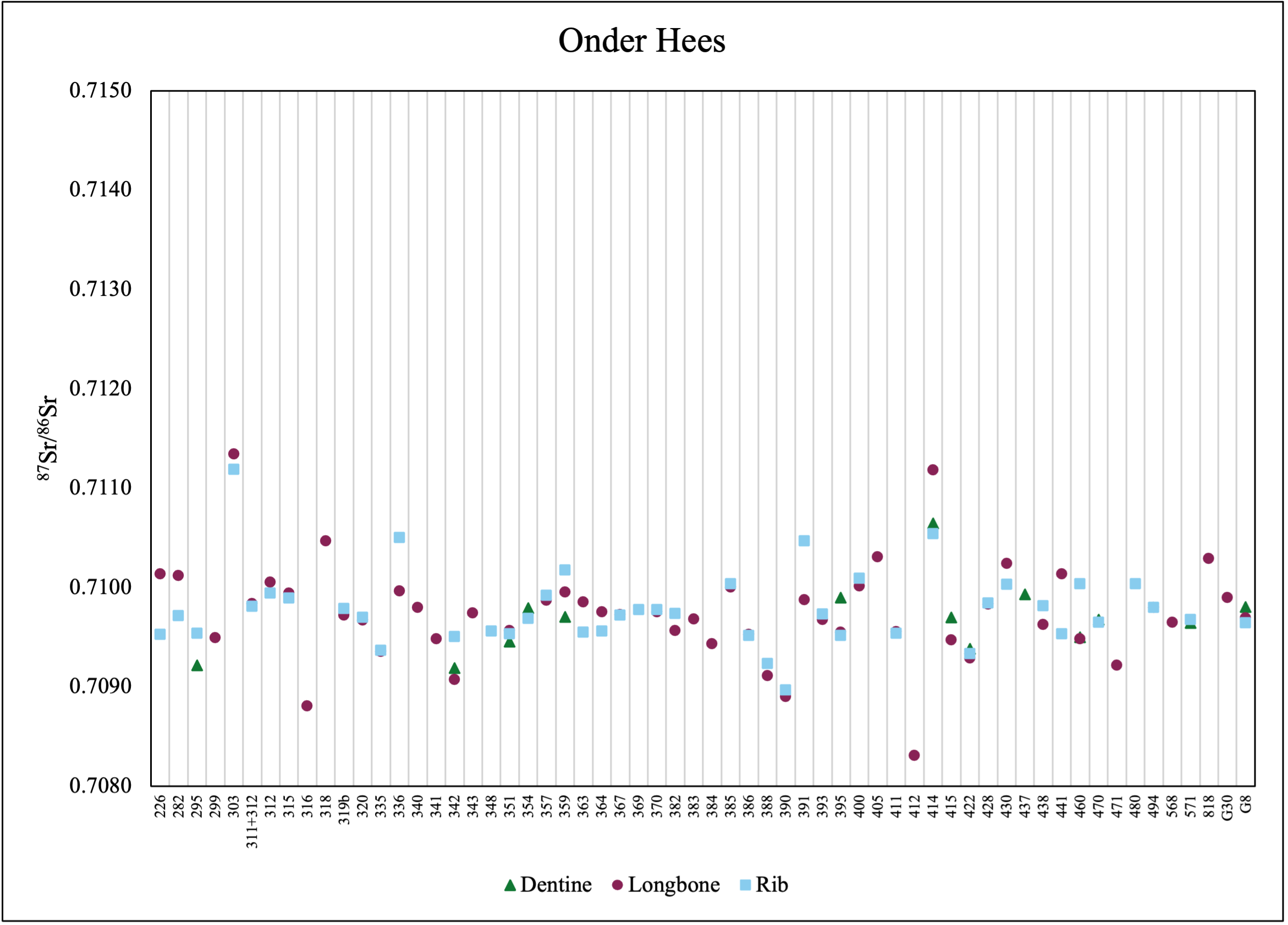


1. **Hengstdalseweg (Hd1-2) [12]**

| **Individual** | **Skeletal element** | **[Sr] in ppm** | **%RSD** | **[Ca] in %** | **%RSD** | **normalized [Sr]** | **^87^Sr/^86^Sr** | **2SE** | **Intra-**  **ind. diff.** |
| --- | --- | --- | --- | --- | --- | --- | --- | --- | --- |
| G1 P2 | Dentine | - | - | - | - | - | 0.713228 | 7 | 0.002565 |
|  | Rib | 187.8 | 0.9 | 46.4 | 0.8 | 162.0 | 0.710663 | 8 |  |
| G5 P11 | Long bone | 145.4 | 0.8 | 37.8 | 0.8 | 154.1 | 0.708628 | 12 | - |
| G7 P14 | Long bone | 251.2 | 0.5 | 48.8 | 1.9 | 206.0 | 0.708866 | 7 | 0.000016 |
|  | Rib | 373.8 | 0.9 | 45.8 | 1.4 | 326.1 | 0.708850 | 8 |  |
| G10 P2 | Long bone | 186.6 | 0.3 | 55.9 | 0.9 | 133.6 | 0.709839 | 8 | 0.000073 |
|  | Rib | 164.8 | 0.9 | 50.1 | 1.1 | 131.6 | 0.709912 | 10 |  |
| G16 P5 | Dentine | 411.1 | 0.4 | 51.7 | 0.2 | 318.1 | 0.710591 | 7 | 0.000301 |
|  | Long bone | - | - | - | - | - | 0.710290 | 12 |  |
| G17 P12 | Dentine | 200.6 | 0.7 | 52.6 | 0.3 | 152.5 | 0.708927 | 7 | 0.000629 |
|  | Long bone | 185.5 | 0.8 | 51.8 | 0.2 | 143.4 | 0.709556 | 8 |  |
| G18 P10 | Long bone | 247.5 | 1.5 | 54.3 | 1.4 | 182.3 | 0.710602 | 7 | - |
| G19 P14 | Long bone | 163.8 | 0.7 | 46.8 | 0.3 | 139.9 | 0.709396 | 6 | - |
| G21 P20 | Dentine | 143.5 | 0.6 | 50.5 | 1.6 | 113.7 | 0.709919 | 8 | 0.000010 |
|  | Long bone | 178.8 | 0.7 | 50.1 | 0.8 | 142.6 | 0.709929 | 7 |  |
| G24 P13 | Long bone | - | - | - | - | - | 0.709904 | 7 | 0.000932 |
|  | Rib | - | - | - | - | - | 0.708972 | 7 |  |
| G25 P10 | Rib | 25.0 | 1.9 | 10.2 | 0.5 | 98.1 | 0.710337 | 7 | - |
| G26 P11 | Long bone | 225.5 | 0.3 | 51.9 | 1.1 | 173.8 | 0.713876 | 8 | 0.000509 |
|  | Rib | 216.7 | 1.5 | 59.3 | 0.2 | 146.1 | 0.714185 | 9 |  |
| G29 P5 | Long bone | 153.5 | 1.4 | 53.5 | 1.6 | 114.7 | 0.713676 | 5 | 0.003993 |
|  | Rib | 115.2 | 0.4 | 48.0 | 0.9 | 95.9 | 0.713576 | 7 |  |
| G30 P9 | Dentine | 184.2 | 0.8 | 51.8 | 1.2 | 142.1 | 0.709460 | 7 | 0.000123  0.000459 |
|  | Long bone | 162.5 | 0.6 | 50.1 | 1.0 | 129.8 | 0.709583 | 10 |  |
|  | Rib | 145.4 | 0.6 | 50.0 | 0.5 | 116.2 | 0.710042 | 7 |  |
| G39 P1 | Long bone | 210.6 | 0.4 | 42.5 | 0.3 | 198.2 | 0.709498 | 7 | - |
| G44 P5 | Dentine | 158.0 | 0.7 | 48.5 | 0.8 | 130.2 | 0.709537 | 6 | 0.000718  0.000155 |
|  | Long bone | 122.0 | 0.4 | 47.8 | 0.9 | 102.0 | 0.710255 | 6 |  |
|  | Rib | 147.3 | 0.5 | 45.0 | 1.2 | 131.0 | 0.710410 | 8 |  |


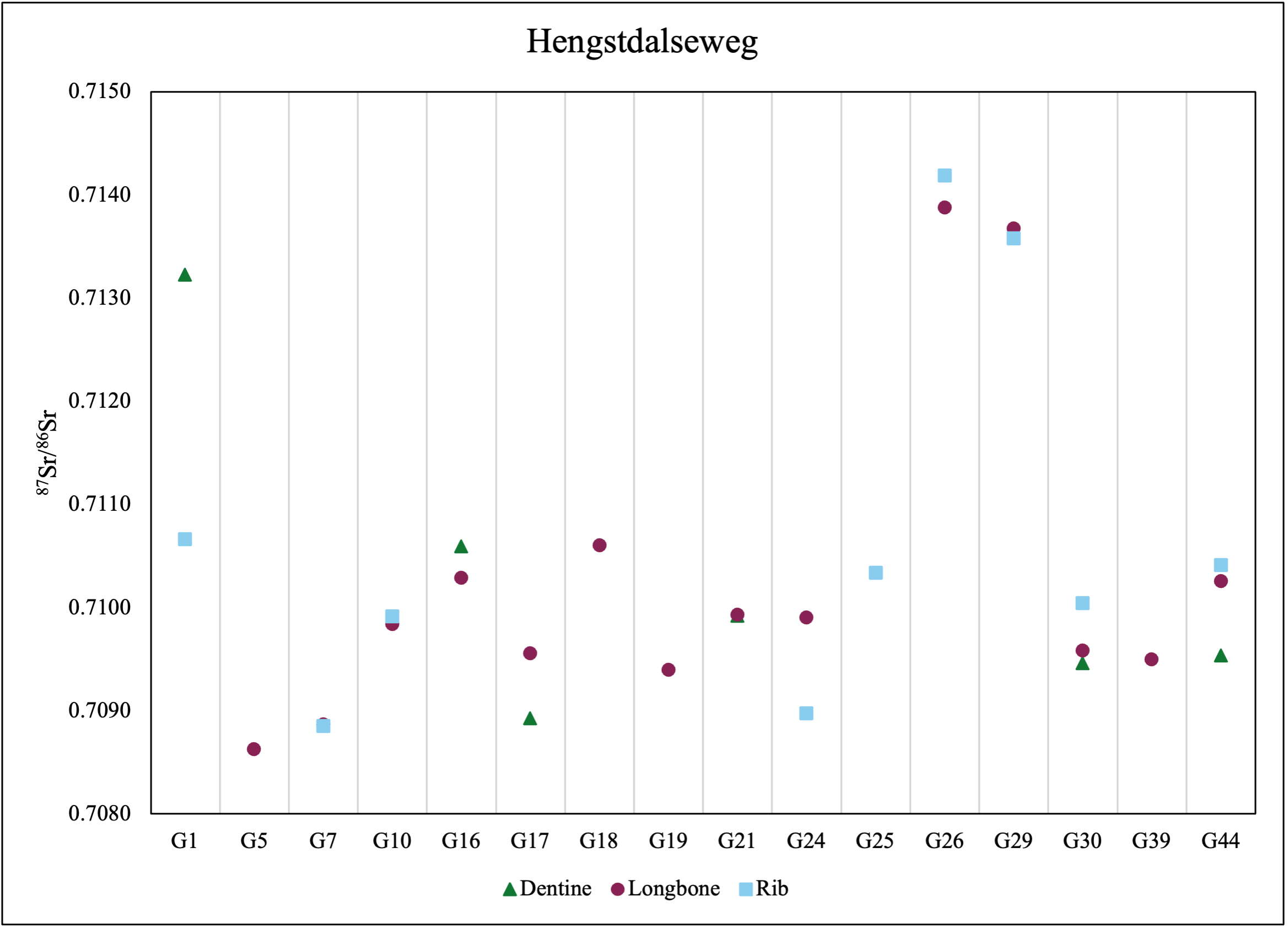


1. **Nijmegen – Kruisweg (Kw1) [12]**

| **Individual** | **Skeletal element** | **[Sr] in ppm** | **%RSD** | **[Ca] in %** | **%RSD** | **normalized [Sr]** | **^87^Sr/^86^Sr** | **2SE** | **Intra-**  **ind. diff.** |
| --- | --- | --- | --- | --- | --- | --- | --- | --- | --- |
| V3 | Dentine | - | - | - | - | - | 0.708603 | 8 | 0.000376  0.000769 |
|  | Long bone | - | - | - | - | - | 0.708979 | 7 |  |
|  | Rib | - | - | - | - | - | 0.709748 | 8 |  |
| V19 | Long bone | - | - | - | - | - | 0.709470 | 8 | 0.000391 |
|  | Rib | - | - | - | - | - | 0.709861 | 12 |  |
| V37 | Rib | - | - | - | - | - | 0.710094 | 7 | - |
| V40 | Dentine | - | - | - | - | - | 0.710829 | 8 | 0.000038 |
|  | Long bone | 113.8 | 0.5 | 49.1 | 1.0 | 92.8 | 0.710791 | 8 |  |
| V45 | Long bone | - | - | - | - | - | 0.708856 | 7 | 0.000054 |
| V45 | Rib | - | - | - | - | - | 0.708802 | 7 |  |
| V62 | Long bone | - | - | - | - | - | 0.711546 | 8 | 0.000520 |
|  | Rib | - | - | - | - | - | 0.711026 | 7 |  |
| V75 | Long bone | - | - | - | - | - | 0.709698 | 6 | - |
| V175 | Rib | - | - | - | - | - | 0.709740 | 7 | - |
| V121 | Long bone | - | - | - | - | - | 0.709857 | 8 | 0.000228 |
|  | Rib | - | - | - | - | - | 0.710085 | 8 |  |
| V126 | Dentine | - | - | - | - | - | 0.708991 | 7 | 0.000172  0.001815 |
|  | Long bone | - | - | - | - | - | 0.708819 | 7 |  |
|  | Rib | - | - | - | - | - | 0.710634 | 7 |  |
| V145 | Long bone | - | - | - | - | - | 0.709608 | 8 | - |


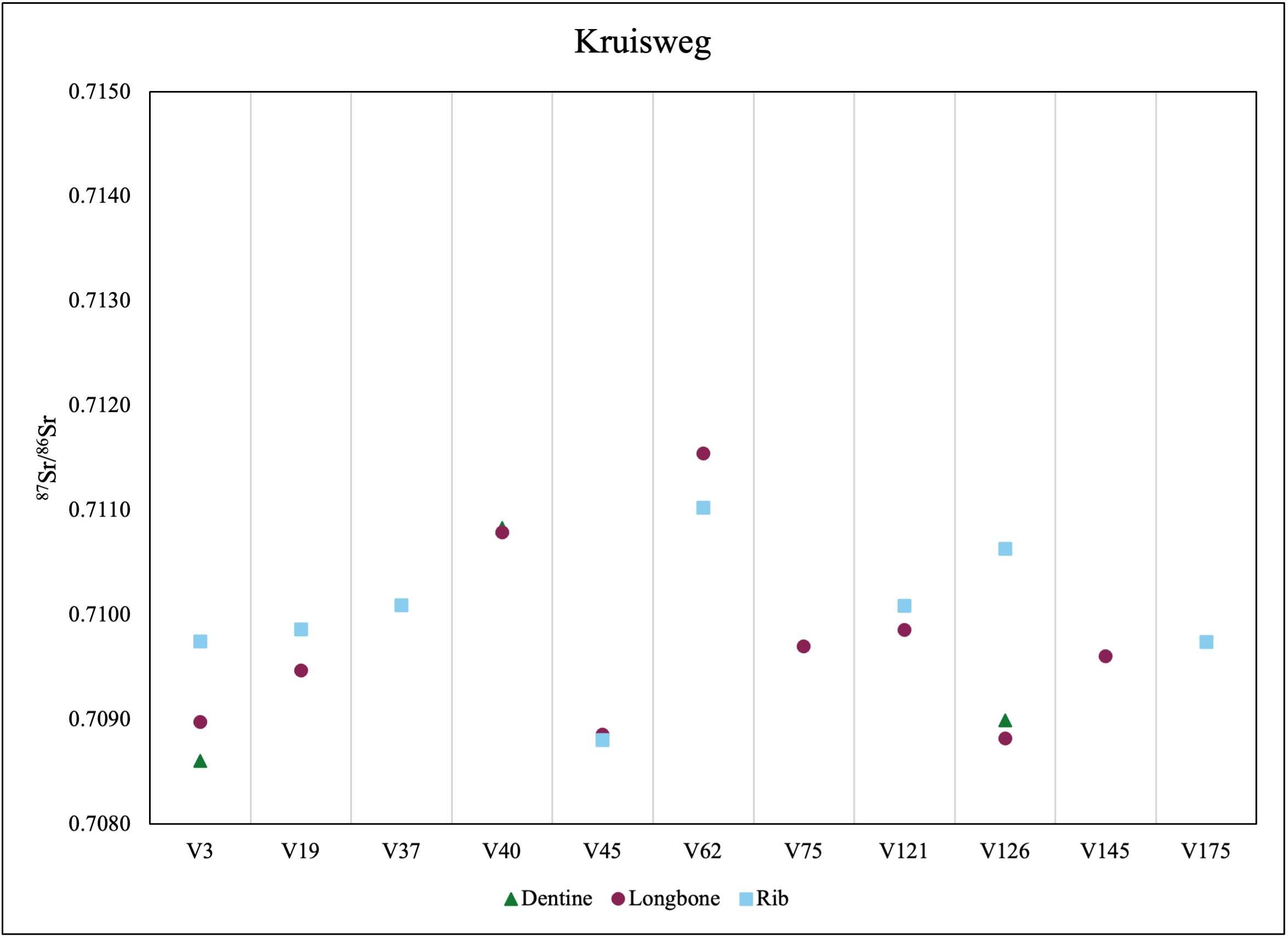


**Bibliography**

1. 1. Hendriks J, Scheeringa-Mulder D. Begraven bij De Eeuwige Lente. Een grafveld uit de vroege ijzertijd aan de Griftdijk in Nijmegen-Noord (project Bo5). Nijmegen: Gemeente Nijmegen; 2026.
2. 2. Van den Broeke PW, Den Braven JA, Daniël AAWJ. Een ijzertijdgrafveld en een erf uit de Ottoonse tijd in Nijmegen-Lent. Archeologisch onderzoek in het Lentseveld. Nijmegen: Gemeente Nijmegen; 2011.
3. 3. Hendriks J, Van Kooten MY. Het bijzondere begrafenisritueel van De Stelt. Een grafveld uit de vroege ijzertijd aan de Steltsestraat in Lent, Nijmegen-Noord (project Sl2). Nijmegen: Gemeente Nijmegen; 2026.
4. 4. Van den Broeke PW, Den Braven JA, Ball EAG. Midden-neolithicum tot en met vroeg-Romeinse tijd in het Zuiderveld – Onderzoek van nederzettingssporen en graven te Nijmegen-Ressen. Nijmegen: Gemeente Nijmegen; 2010.
5. 5. Eimermann E, Van den Broeke PW. Een grafveld uit de ijzertijd en sporen uit andere perioden in Nijmegen-Noord. Proefsleuvenonderzoek in het Zuiderveld; project Zn3. Nijmegen: Gemeente Nijmegen; 2017.
6. 6. Chariots on Fire, Reins of Power: Early La Tène elite burials from the Lower Rhine-Meuse region and their Northwest European context. Leiden: Sidestone Press; 2024.
7. 7. Roymans N, Van den Dikkenberg L, Kootker LM. Societal change and interregional connectivity in the 5th-century BC Lower Rhine-Meuse region. In: Roymans N, Theunissen L, Swinkels L, Van der Vaart-Verschoof S, editors. Chariots on Fire, Reins of Power: Early La Tène elite burials from the Lower Rhine-Meuse region and their Northwest European context. Leiden: Sidestone Press; 2024. p. 481-524.
8. 8. Daniël AAWJ. Germaanse invloeden in Nijmegen-Noord? Aanvullingen op het grafveld uit de midden-Romeinse tijd op ’t Klumke. Twee opgravingen in plangebied Grote Boel, gemeente Nijmegen; projecten Ngk7 en Ngk9. Nijmegen: Gemeente Nijmegen; 2020.
9. 9. Hendriks J. Het Romeinse grafveld ‘Rust Wat’. Archeologisch onderzoek aan de Griftdijk in Nijmegen-Noord (project Bo6/8), provisional title. Nijmegen: Gemeente Nijmegen; in prep.
10. 10. Van de Geer P. Een Romeins grafveld in Broodkorf-Noord. Proefsleuvenonderzoek op vindplaatsen 64, 117 en 118 in Koudenhoek, te Lent – gemeente Nijmegen. Nijmegen: Gemeente Nijmegen; 2021.
11. 11. Koster A. The cemetery of Noviomagus and the wealthy burials of the municipal elite. Nijmegen: Museum Het Valkhof; 2013.
12. 12. Timmerman E. De Romeinse grafvelden aan de Kruisweg en Hengstdalseweg in Nijmegen-Oost (provisional title). Nijmegen: Radboud Universiteit; in prep.
